# Low-dimensional morphospace of topological motifs in human fMRI brain networks

**DOI:** 10.1162/netn_a_00038

**Published:** 2018-06-01

**Authors:** Sarah E. Morgan, Sophie Achard, Maite Termenon, Edward T. Bullmore, Petra E. Vértes

**Affiliations:** Brain Mapping Unit, Psychiatry Department, Cambridge University, Cambridge, United Kingdom; Cambridgeshire and Peterborough NHS Foundation Trust, Huntingdon, PE29 3RJ, UK; Cambridgeshire and Peterborough NHS Foundation Trust, Huntingdon, PE29 3RJ, UK; Brain Mapping Unit, Psychiatry Department, Cambridge University, Cambridge, United Kingdom; Cambridgeshire and Peterborough NHS Foundation Trust, Huntingdon, PE29 3RJ, UK; ImmunoPsychiatry, Immuno-Inflammation Therapeutic Area Unit, GlaxoSmithKline R&D, Stevenage, SG1 2NY, UK; Brain Mapping Unit, Psychiatry Department, Cambridge University, Cambridge, United Kingdom

**Keywords:** Morphospace, Network motifs, Graph theory, fMRI, Functional connectivity, Human brain networks

## Abstract

We present a low-dimensional morphospace of fMRI brain networks, where axes are defined in a data-driven manner based on the network motifs. The morphospace allows us to identify the key variations in healthy fMRI networks in terms of their underlying motifs, and we observe that two principal components (PCs) can account for 97% of the motif variability. The first PC of the motif distribution is correlated with efficiency and inversely correlated with transitivity. Hence this axis approximately conforms to the well-known economical small-world trade-off between integration and segregation in brain networks. Finally, we show that the economical clustering generative model proposed by Vértes et al. (2012) can approximately reproduce the motif morphospace of the real fMRI brain networks, in contrast to other generative models. Overall, the motif morphospace provides a powerful way to visualize the relationships between network properties and to investigate generative or constraining factors in the formation of complex human brain functional networks.

## Author Summary

Motifs have been described as the building blocks of complex networks. Meanwhile, a morphospace allows networks to be placed in a common space and can reveal the relationships between different network properties and elucidate the driving forces behind network topology. We combine the concepts of motifs and morphospaces to create the first motif morphospace of fMRI brain networks. Crucially, the morphospace axes are defined by the motifs, in a data-driven manner. We observe strong correlations between the networks’ positions in morphospace and their global topological properties, suggesting that motif morphospaces are a powerful way to capture the topology of networks in a low-dimensional space and to compare generative models of brain networks. Motif morphospaces could also be used to study other complex networks’ topologies.

## INTRODUCTION

Understanding the factors influencing the global topology of fMRI brain networks is a long-held ambition of network neuroscience. The energetic cost of forming and maintaining long-distance connections has long been established as an important factor shaping brain networks (Bullmore & Sporns, 2012; Ramon y Cajal, 1995; Young, 1992); for example, as early as 1899 Ramón y Cajal wrote about the importance of conservation of time, space and material in determining neuronal morphology and connections, (see chapter 5 of Ramon y Cajal, 1995). However, it is also well known that cost minimization alone cannot explain all observed network features (Kaiser & Hilgetag, 2006). In 2012, Vértes et al. showed that several properties of fMRI networks can be reproduced by a generative model that encodes a trade-off between the cost of long-distance connections and the topological features they enable (Vértes et al., 2012). Generative models typically focus on a number of global metrics to characterize the similarity between the observed and modelled network topology, for example, global efficiency or modularity (Klimm, Bassett, Carlson, & Mucha, 2013; Rubinov & Sporns, 2010). However, the plethora of metrics available can make results difficult to compare across studies, metrics often overlap (Li, Wang, de Haan, Starn, & Van Mieghem, 2011), and a top-down approach risks missing functionally important features. Important questions remain. For example, what are the key topological features of fMRI networks, which a generative model should capture? What are the relationships between different network metrics in fMRI networks?

We present a data-driven approach using motifs to characterize brain networks, removing the need for arbitrarily chosen global metrics. Motifs are the building blocks of networks and are known to vary between networks with different topologies (Milo et al., 2004, 2002). They have also been used to classify networks into superfamilies, with different motif fingerprints shaped by different functional roles (Milo et al., 2004). In 2004, Sporns and Kötter showed that motifs can give insight into the structure and function of brain networks (Sporns & Kötter, 2004). Their results suggested that at the neuronal level, brain networks optimize the number of functional states available to support efficient integration of information. Certain motifs have also been found to play important roles in brain network dynamics; sustaining network activity (Garcia, Lesne, Hilgetag, & Hütt, 2014) and maintaining or disrupting network synchronization, which is believed to be important for many cognitive processes (Gollo & Breakspear, 2014; Gollo, Mirasso, Sporns, & Breakspear, 2014). Overall, (a) the relationship between a network’s motif fingerprint and its function, which has been observed in a wide variety of biological and man-made networks, (b) the important roles motifs play in neuronal networks’ structure and dynamics, and (c) their ability to characterize a network in terms of its basic building blocks motivate the question: Can motifs be used to map the space of macroscopic fMRI brain networks?

To answer this question, we begin by characterizing our networks in terms of their motif fingerprints. We then perform a principal component analysis (PCA) and find that two principal components (PCs) can explain 97% of the motif variability. This allows us to build a low-dimensional morphospace defined by these first two PCs. Morphospaces have already been shown as a promising approach to study network topology (Avena-Koenigsberger, Goñi, Solé, & Sporns, 2014; Goñi et al., 2014). Work by Pearcy et al. (Pearcy, Crofts, & Chuzhanova, 2015) has also used network motifs to obtain a low-dimensional morphospace of metabolic networks. To our knowledge, here we present the first morphospace of fMRI brain networks whose axes are based on a principal component analysis of motif fingerprints.

In the following, first we show that motifs allow the global topological properties of fMRI brain networks to be described by a low-dimensional space. PC1 represents the small-worldness axis and correlates with the networks’ global efficiency and transitivity, while PC2 correlates with the networks’ assortativity. These correlations between the motif PCs and the global metrics are interesting because they highlight the motifs’ ability to capture the topology of complex networks. This nontrivial result also allows us to map the topology of the networks in a data-driven manner, without needing to select which network metrics to study. In the future, we expect this powerful approach to be useful for mapping other types of networks, both in the brain and in other complex systems.

Second, we suggest a tentative link between the networks’ topology and their spatial embedding. In particular we find some evidence that PC1 (the small-worldness axis) correlates with the Euclidean distance of the longest network edges. This result was not reproduced in a second (smaller) dataset and we highlight it as needing further confirmation in other large datasets.

The fact that 97% of the variability in the motif fingerprints can be explained by just two principal components and that the motif PCs correlate with global topological metrics suggests that the networks lie in a low-dimensional topological space. This result is in line with prior research suggesting that very few parameters are needed to reproduce key global topological features of macroscopic brain networks, for example results from generative models (Betzel et al., 2016; Klimm et al., 2013; Vértes et al., 2012). Our identification of PC1 as the small-worldness axis raises the question of which aspects of the motif fingerprints and morphospace a simple generative model that takes into account the economic trade-off between long-distance edges’ cost and their useful topological properties can reproduce. One advantage of generative models is that the model parameters can be changed manually, allowing us to also explore the effect of topological and distance parameters separately. In the final part of this work we show that the economical clustering model proposed by Vértes et al. (2012) can largely reproduce the motif fingerprints and morphospace obtained experimentally and that both the topological and distance parameters in this model influence the networks’ positions along both PCs. Interestingly, other plausible models (e.g., the economical preferential attachment model) are unable to do this; hence our motif morphospace provides a simple and powerful way to discriminate between models and evaluate model data fit.

## RESULTS

### Generation of the Morphospace From Motif Fingerprints

**Figure 1.**
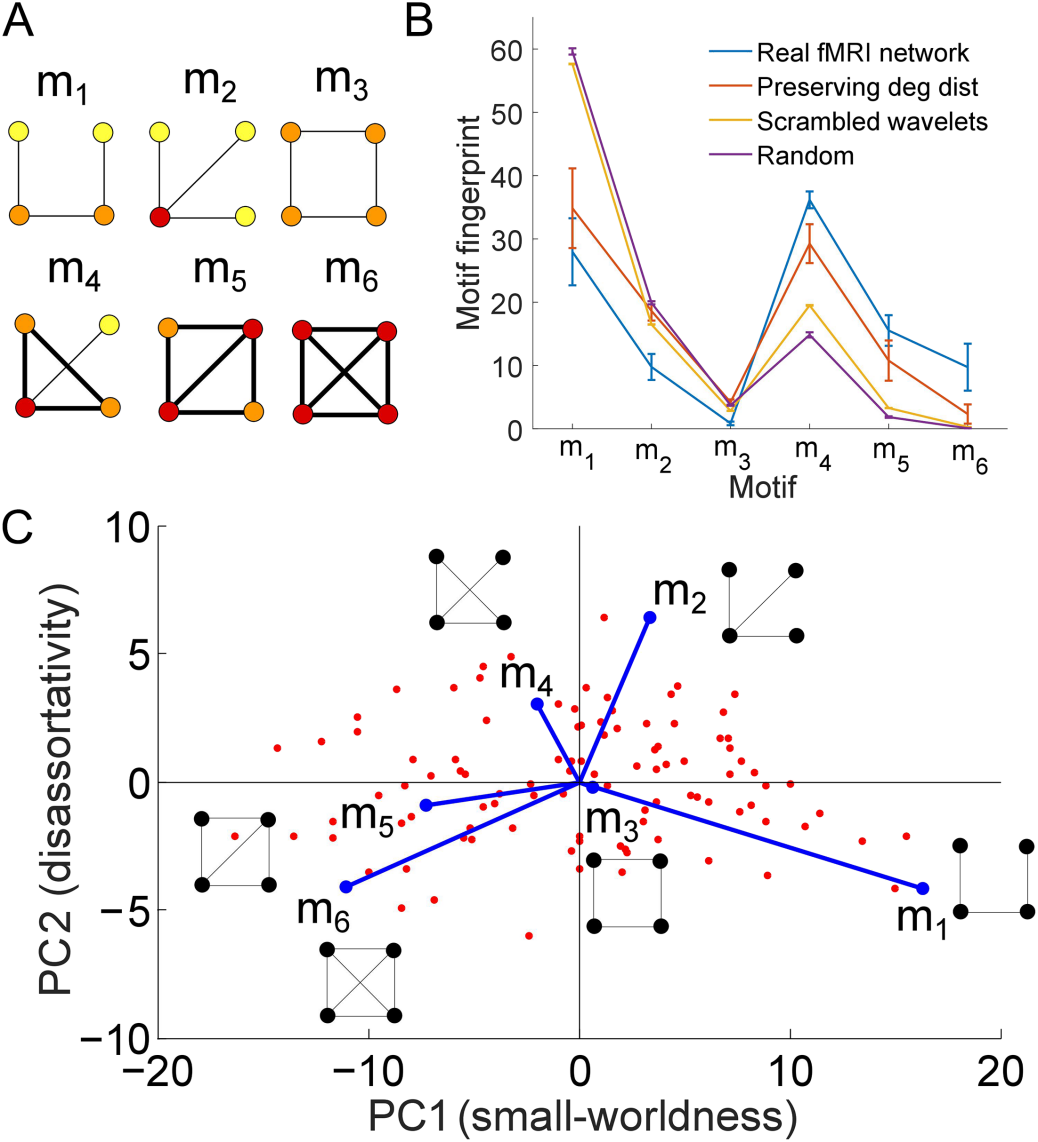
Motifs, motif fingerprints, and motif morphospace. (A) All six possible four-node undirected motifs. Motif nodes are colored according to their degree, where yellow nodes have degree 1, orange have degree 2, and red have degree 3. Motif edges forming triangles are highlighted in bold. (B) Motif fingerprint averaged across all subjects (blue). The error bars show ±*σ*, where *σ* is the standard deviation. The motif fingerprint from three different null models are shown for comparison, namely randomized networks not preserving the degree distribution (purple), randomized networks preserving the degree distribution (red), and randomized networks obtained by scrambling the wavelet coefficients (orange). In the last two cases results were averaged over 100 realizations per real fMRI network. (C) The motif PC morphospace plotted using the first two PCs. Here each red dot represents an individual subject, plotted in PC space. The original motifs are also plotted as vectors in the PC space. The axes are labeled as small-worldness and disassortativity due to the correlations of PC1 and PC2 with global network metrics.

We begin by calculating undirected fMRI networks for 100 healthy subjects from the Human Connectome Project by correlating the regional fMRI wavelet time series and thresholding the resulting correlation matrices. Details of the data and the preprocessing are given in the Methods section. Unless otherwise stated, we take a threshold of 800 edges (a connection density of ≈ 20%). We then characterize the networks in terms of their motifs. G′ = (V′, E′) is a motif of the graph G = (V, E) with vertices V and edges E, if V′ ⊆ V and E′ is a subset of E such that the vertices of each edge in E′ are in V′. In this work we consider induced motifs, for which E′ includes all edges of G that end on vertices V′ (Diestel, 2006). We focus on the six possible four-node, undirected motifs, which are shown in Figure 1A. We chose to study four-node motifs because there are only two possible three-node undirected motifs, which greatly limits the diversity of connectivity patterns that can be studied. The six possible four-node motifs provide much greater variety. As discussed below and in the Supplementary Information (Morgan, Achard, Termenon, Bullmore, & Vértes, 2018), five-node motifs gave similar results to four-node motifs. Following Sporns & Kötter (2004), these motifs can be denoted as $m_{i}^{4}$, where *i* ∈ 1, 6. Since we only consider four-node motifs, we drop the superscript 4 for brevity. We note that the different motifs have different numbers of edges and closed triangles; *m*_6_ is fully connected, while *m*_1_ and *m*_3_ have the least edges and no closed triangles. Different motifs also exhibit different levels of degree heterogeneity, as shown by the color of the nodes in Figure 1A; *m*_3_ and *m*_6_ have nodes with the most homogeneous degrees, while *m*_2_ and *m*_4_ have nodes with the most heterogeneous degrees.

For each individual network, we count the number of each of these motifs and normalize with respect to the total number of motifs in the network. This gives us a motif fingerprint for each network; further details are given in the Methods section. The average fingerprint across all networks is shown in Figure 1B, alongside the standard deviation across the subjects and the motif fingerprints for three randomized networks for comparison. In particular, we consider randomized networks that do not preserve the degree distribution, randomized networks that do preserve the degree distribution, and randomized networks in which the wavelet coefficients are scrambled before being correlated with each other, as proposed by Zalesky, Fornito, & Bullmore (2012; see the Methods section for details of all of these null models).

Randomized networks that do not preserve the degree distribution are unable to reproduce any of the motif scores of the original fMRI brain networks to within one standard deviation. In the real networks, we observe higher proportions of *m*_4_, *m*_5_, and *m*_6_ compared with these fully randomized networks and lower proportions of *m*_1_, *m*_2_, and *m*_3_. Unsurprisingly, randomized networks preserving the degree distribution are able to reproduce more of the motif fingerprint than randomized networks that do not preserve the degree distribution, although even in this case only two motifs exhibit mean fingerprint scores that are within one standard deviation of the real fMRI results. Motifs *m*_4_ and *m*_6_ again exhibit higher proportions in the real fMRI networks than in the randomized results, while *m*_2_ and *m*_3_ exhibit lower proportions. Hence the degree distribution has an influence on the motif fingerprint, as might be expected, but it is not the only important factor. The final null model is designed to account for the fact that the usual estimators of correlation are topologically biased and can induce spurious topological features into the network architecture; for example, they tend to exhibit more closed triangles than random Erdős-Rényi networks (Zalesky et al., 2012). This null model gives a motif fingerprint that is closer to the real fMRI results than randomized networks that do not preserve the degree distribution, as might be expected, but again does not reproduce any of the motif scores to within one standard deviation. The inability of the randomized networks with scrambled wavelet coefficients to reproduce the motif fingerprint shows that the motif fingerprint is not solely determined by the topological bias inherent in estimating correlations.

We perform a PCA on the motif fingerprints for all 100 subjects, again as described in the Methods section. The first PC explains 86% of the total variability, and the first two PCs explain 97% of the total variability; see the cumulative variability plot shown in Supplementary Figure 1 (Morgan et al., 2018). Note that here we use four-node motifs, however, similar results can be found using the 21 possible five-node motifs (see section 6 of the Supplementary Information; Morgan et al., 2018). Hence we focus on the first two motif PCs. By plotting the individual networks as a function of these PCs, we can create a two-dimensional morphospace, as shown in Figure 1C (here individual subjects are represented by red dots). In order to visualize the extent to which different motifs underlie the PCs, we also plot the original motif fingerprint variables as vectors in the PC space (sometimes known as a biplot). We find that PC1 is driven by a difference between the “chain-like” *m*_1_ and the more densely clustered *m*_5_ and *m*_6_. Networks with high PC2 values have a higher proportion of *m*_2_ and *m*_4_, which have greater variability in motif node degree than the other motifs. Remarkably, using five-node motifs produces a very similar biplot to the four-node motifs, with a similar pattern of motifs underlying the morphospace, as shown in Supplementary Figure 7B (Morgan et al., 2018). We use a threshold of 800 edges (≈ 20% connection density), however similar motif fingerprints and biplots are obtained with 400 and 1,200 edges (≈ 10% or 30% connection density); see section 2 of the Supplementary Information (Morgan et al., 2018). We can also reproduce the results using a different parcellation (the AICHA parcellation [Joliot et al., 2015], with 384 regions rather than 90), as well as in the HCP retest dataset and in an independent dataset, see sections 3, 4, and 5 of the Supplementary Information (Supplementary Information; Morgan et al., 2018), respectively.

### Relationship Between the PCs and Global Network Metrics

**Figure 2.**
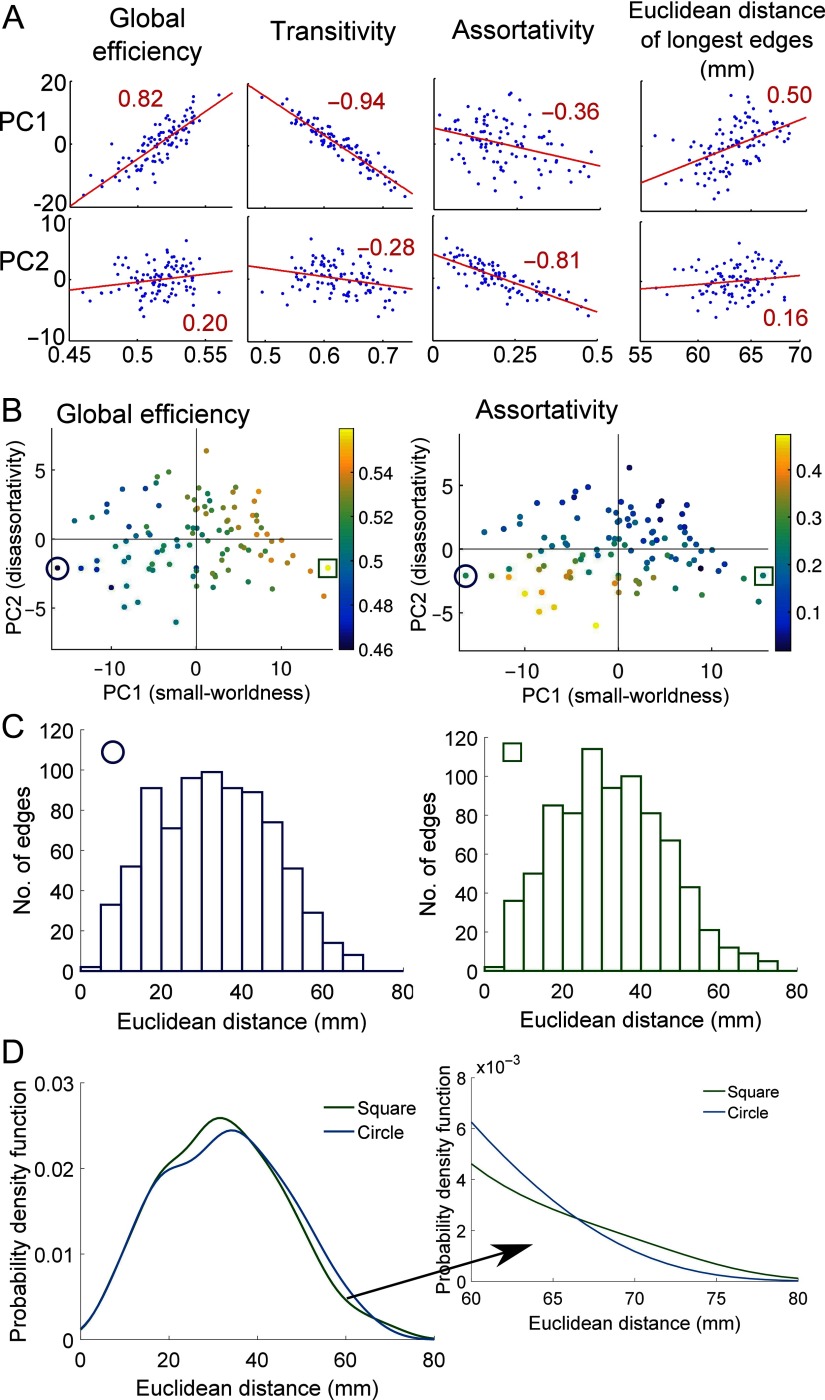
Motif morphospace dimensions and global topology. (A) The correlation between the first two PCs and the global efficiency, assortativity, and transitivity of the networks. The correlation between the PCs and the average Euclidean distance of the longest 5% of edges is also shown. In all plots, the Pearson correlation coefficient is shown in red. (B) The motif morphospace (as shown in Figure 1C), colored according to the global efficiency and the assortativity of each network. (C) Edge distance distributions for the subject with the highest PC1 score (denoted by a green square) and the subject with the lowest PC1 score (denoted by a blue circle). (D) The probability density functions for the two subjects (fitted using a kernel distribution) and a zoom in on the longest Euclidean distances.

To investigate the differences in the networks that underlie the PC space further, we calculate the Pearson correlations between the PCs and global network metrics, namely global efficiency, assortativity, and transitivity. The correlations are summarized in Figure 2A, and in Figure 2B we plot the PC space colored according to the global efficiency and assortativity. With the exception of a weak correlation between PC3 and global efficiency (with Pearson correlation coefficient *r* = −0.22, and a *p* value for a two-tailed Pearson correlation test using the student’s *t* distribution of *p* = 0.03), PCs 3, 4, 5, and 6 did not correlate significantly (*p* > 0.05) with any of the global network measures tested. This is as expected because 97% of the motif variability is explained by PCs 1 and 2.

PC1 is positively correlated with global efficiency (with a Pearson correlation coefficient *r* = 0.82, *p* < 0.001) and negatively correlated with transitivity (*r* = −0.94, *p* < 0.001). Hence as the proportion of the “chain-like” *m*_1_ increases and the proportion of the fully clustered *m*_6_ decreases, the global efficiency of the networks tends to increase, while the transitivity decreases. This inverse relationship between global efficiency and transitivity is reminiscent of the trade-off navigated by small-world networks, which can exhibit both high clustering and high global efficiency (Watts & Strogatz, 1998). FMRI brain networks are known to exhibit small-world properties (Bassett & Bullmore, 2006, 2016), and our results provide further evidence that this is a key axis of variability in their topologies. In agreement with these results, PC1 correlates with the small-worldness coefficient of the networks as defined by Humphries, Gurney, and Prescott (2006) (*r* = 0.87, *p* < 0.001; see Supplementary Figure 8, Morgan et al., 2018). Hence we label PC1 as small-worldness. We note that Stam and van Straaten (2012) suggested a heuristic model of brain networks (Figure 10 in their paper) in which one of the main axes is defined by small-worldness and is similar to our first PC.

PC2 is most strongly correlated with assortativity (*r* = −0.81, *p* < 0.001), hence networks with higher proportions of *m*_2_ and *m*_4_ tend to have lower assortativity values and we label PC2 as disassortativity. The observation that apical motifs (e.g., *m*_2_) tend to have high PC2 scores suggests that PC2 might also be expected to correlate with hierarchy. The measure of hierarchy proposed by Mones, Vicsek, and Vicsek (2012), which measures the heterogeneity in closeness centrality of the network’s nodes, does correlate strongly with PC2 (*r* = 0.74, *p* < 0.001) and more weakly with PC1 (*r* = −0.41, *p* < 0.001). Ravasz and Barabasi (2003) studied hierarchical modularity using the scaling of the nodes’ clustering coefficients with their degrees. The exponent of this relationship correlates most strongly with PC1 (*r* = 0.63, *p* < 0.001) and less strongly with PC2 (*r* = 0.40, *p* < 0.001). This difference reflects the fact that Ravasz’s approach is more sensitive to the networks’ modularity than Mones’ measure. PC2 is not dissimilar to Stam’s vertical axis, which he labels diversity (Stam & van Straaten, 2012), and we note that from Figure 1C, motifs that show high PC2 values tend to have a higher level of node degree diversity than motifs with low PC2 values.

We note that the correlations of PC1 with the efficiency and transitivity of the networks and PC2 with the assortativity described above are extremely robust. They can be replicated with 400 or 1,200 edges instead of 800, as shown in section 2 of the Supplementary Information (Morgan et al., 2018). The results are robust to changing the parcellation to the AICHA parcellation (Joliot et al., 2015), which has 384 regions rather than 90; see section 3 of the Supplementary Information (Morgan et al., 2018). The results can also be reproduced both in the retest HCP dataset and the independent Cambridge dataset described in the Methods section. For details, see sections 4 and 5 of the Supplementary Information (Morgan et al., 2018). Finally, if the global metrics (efficiency, transitivity, and assortativity) are included directly in the PCA in addition to the motif fingerprints, the directions of the efficiency, transitivity, and assortativity vectors in the resulting biplot are broadly the same as the directions of the corresponding correlations shown in Figure 2. These results are shown in section 8 of the Supplementary Information (Morgan et al., 2018), alongside the PCA loadings before and after including the global metrics.

### Relationship Between the PCs and the Spatial Embedding of the Networks

Having observed significant correlations between the PCs and global network metrics, we now turn to the relationship between the PCs and the spatial embedding of the networks. We take the Euclidean distance between the brain regions’ centroids as a measure characterizing their spatial embedding. We begin by calculating the correlation between PCs 1 and 2 and the total Euclidean distance of each network’s connections and obtain *r* = 0.03 (*p* = 0.75) and *r* = 0.17 (*p* = 0.09), respectively. We conclude that there is no significant correlation between total Euclidean distance and PCs 1 and 2. Note that there is also no significant correlation (*p* > 0.05) between the global efficiency, assortativity, or transitivity and the total Euclidean distance.

However, while there is no correlation between the PCs and the total Euclidean distance, we do observe significant correlation between PC1 and the average Euclidean distance (“length”) of the longest 5% of edges in each network, with *r* = 0.50 (*p* < 0.001), as shown in Figure 2A. In contrast, there is no significant correlation between the length of the longest 5% of edges and PC2 (*r* = 0.16, *p* = 0.11). To calculate the distance of the longest 5% of edges, we rank the edges in an individual network by the Euclidean distance between the centroids of the regions they connect. We then select the 5% of edges that have the greatest Euclidean distances and calculate the mean of their Euclidean distances. The positive correlation we observe with PC1 means that the longest 5% of edges tend to be longer in networks with a relatively high proportion of “chain-like” *m*_1_ (which from Figure 1 also tend to be those with higher global efficiency). In Figure 2C, we plot the distance distribution for the subjects with the highest and lowest PC1 values, represented by a square and a circle respectively in Figure 2B. As expected, the distance distribution for the subject with the highest PC1 value has a longer tail at long distances (for the subject denoted by a square the average Euclidean distance of the longest 5% of edges was 63.2 mm, compared with 61.7 mm for the subject denoted by a circle). An alternative approach to testing for a relationship between the PCs and the “tailedness” of the distance distributions is to measure their kurtosis. In agreement with our results from the distance of the longest edges, we find that the kurtosis of the Euclidean distance distribution correlates with PC1 (*r* = 0.39, *p* < 0.001) and weakly with PC2 (*r* = −0.24, *p* = 0.02). In other words, networks whose edges’ Euclidean distance distribution exhibits a larger kurtosis have higher PC1 scores, in keeping with the observation that networks whose longest edges have greater Euclidean distances have higher PC1 scores.

In order to understand the difference between the distribution of the longest edges in networks with high and low PC1 scores in more depth, in section 9 of the Supplementary Information (Morgan et al., 2018) we plot the longest 5% of edges from our two example subjects on the brain. We observe that the long-distance edges in the subject represented by a circle (whose longest edges are shorter on average) are more likely to be interhemispheric, while the subject represented by a square (whose longest edges are longer on average) are more likely to be intrahemispheric, often connecting the front and the back of the brain. To explore this finding further in the whole dataset, we count the number of the longest 5% of edges in each subject that are interhemispheric. The results correlate negatively with PC1 (*r* = −0.44, *p* < 0.001); see Supplementary Figure 12 (Morgan et al., 2018). In other words, the longest edges in networks with high PC1 scores (which tend to have high global efficiency) are more likely to be intrahemispheric than the longest edges in networks with low PC1 scores. These edges tend to connect the front and the back of the brain and could therefore be particularly important for brain integration. Importantly, there is no correlation between PC1 and the total number of interhemispheric edges in the network, hence on average networks with low or high PC1 scores have the same number of inter (and therefore intra) hemispheric edges in total.

The significant correlation between PC1 and the length of the longest edges is robust to changing the percentage of the longest edges considered from 1% to 10% and to considering 400 or 1,200 edges instead of 800 (see sections 2 and 9 of the Supplementary Information (Morgan et al., 2018). We also reproduce the result in the retest HCP dataset and obtain *r* = 0.43 (*p* < 0.001). Interestingly, the result is not significant (*r* = 0.30, *p* = 0.14) in an independent dataset with 26 subjects (the Cambridge dataset) as discussed in section 5 of the Supplementary Information (Morgan et al., 2018; note that the kurtosis of the distributions also does not significantly correlate with the PCs in this case). This may be due to the reduced number of subjects available, however further work is required to clarify the reason for this difference. We include this tentative result here because we believe it is important for the community to test in other large datasets.

### Comparison With Generative Models

The motif morphospace described above enables us to map the global topology of fMRI brain networks and to explore the relationships between different global properties of the networks. Generative models provide a way to study the driving forces underlying network topology, and previous work has suggested that a simple two-parameter generative model can capture many of the global features of fMRI brain networks (Vértes et al., 2012). Hence in the final part of this work we investigate the extent to which a simple two-parameter generative model can capture the motif morphospace we propose.

**Figure 3.**
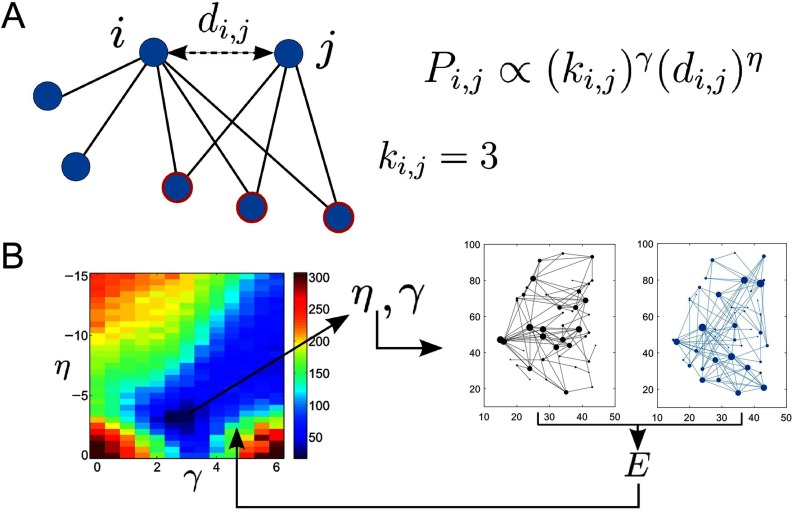
Schematic of economical clustering generative model. (A) The probability of a new link being added between nodes *i* and *j* depends on both the Euclidean distance between them (*d*_*i*,*j*_) and the number of nearest neighbors they have in common (*k*_*i*,*j*_ = 3 in this example). (B) Illustration of the generative model steps. Networks are generated at a range of different *η* and *γ* values and their fit to the real brain networks is calculated via an energy function, E, which compares networks on the basis of global efficiency, modularity, mean clustering coefficient, and degree distribution (as used by Vértes et al., 2012). On the left-hand side the energy is plotted as a function of *η* and *γ*. On the right-hand side, an example real brain network and generated network are shown for illustration (in black and blue, respectively). The final generated networks are simulated using the optimized values of *η* and *γ*.

Vértes et al. (2012) found that the economical clustering generative model given by Equation 1 was able to reproduce many aspects of fMRI brain network topology. This generative model encapsulates the trade-off between the cost of long-distance connections and the network’s topology. Figure 3A illustrates the factors that determine the probability of connecting nodes *i* and *j* (*P*_*i*,*j*_) in the model. The length of the connection between nodes *i* and *j* is approximated by their Euclidean distance, *d*_*i*,*j*_, and the probability of connecting the nodes depends on this distance and the parameter *η*. The topological term employed by the model sets the probability of connecting nodes *i* and *j* to increase with the number of nearest neighbors they have in common, *k*_*i*,*j*_. The strength of this dependence is given by the parameter *γ*. Overall, *P*_*i*,*j*_ is given by

$$P_{i,j}\propto {\left(k_{i,j}\right)}^{\gamma }{\left(d_{i,j}\right)}^{-\eta }.$$ (1)

We use the economical clustering model to simulate the topology of our fMRI networks. All of the results described up to this point are robust to thresholding the networks at either 400, 800, or 1,200 edges (≈ 10%, 20%, or 30% connection density). However, the generative models require relatively sparse networks, therefore we use a threshold of 400 edges in this section. We also use a single hemisphere (left) rather than both hemispheres, as in Vértes et al. (2012) and Betzel et al. (2016).

We use an independent dataset and an identical approach to generating the networks as Vértes et al. (2012). We determine the parameters (*γ* and *η*) by exploring the parameter space manually to obtain values of *γ* and *η* that optimize the energy function used by Vértes et al. (2012), which is calculated from the global efficiency, modularity, mean clustering coefficient, and degree distribution of the generated networks in comparison to the real HCP fMRI brain networks. For our networks, which have fewer nodes than those used in Vértes et al. (2012), we find that *η* = −3 and *γ* = 2.5 are the optimal values; see section 10.1 of the Supplementary Information (Morgan et al., 2018) for more details. Note that while these parameters were optimized for the measures listed above, they were not optimized to match the motif fingerprints or other network measures including the assortativity. Here we do not address the question of which network properties are required and which could be by-products of other properties, although we note that this is a current question in the field (Rubinov, 2016) and an interesting avenue for future research. We also stress that the real fMRI networks were only used to optimize *η* and *γ* and the simulated networks were generated with no other knowledge of the real networks. The process is illustrated in Figure 3B.

**Figure 4.**
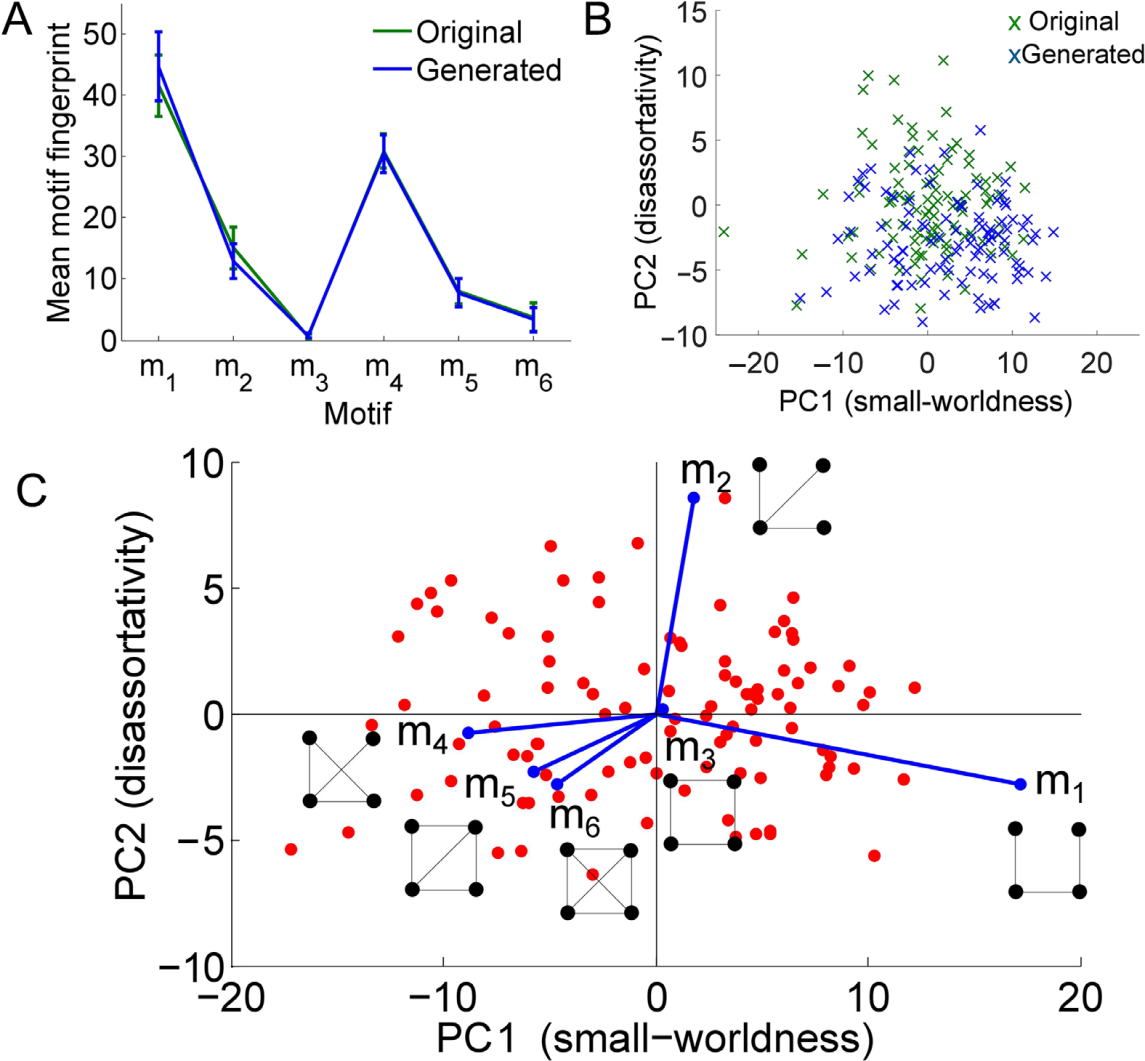
Generative model results. (A) The motif fingerprints of the original real brain networks (with a single hemisphere and 400 edges), alongside the motif fingerprints of the generated networks (*η* = −3 and *γ* = 2.5). (B) The generated networks projected onto the space of the original networks. (C) The biplot of the generated networks.

In Figure 4A, we plot the average motif fingerprint of the generated networks alongside the real fMRI brain networks and note that they are the same to within ±*σ*, the standard deviation. When we project the generated networks onto the original morphospace of fMRI brain networks, we find that they lie in a similar region, see Figure 4B. In Figure 4C, we plot the biplot of the generated networks, which is remarkably similar to the biplot shown in Figure 1C. The correlation between the PCs and the global efficiency, assortativity, and transitivity are similar to those obtained for the real fMRI brain networks; see the Supplementary Information (Morgan et al., 2018) for details. The correlation between the average Euclidean distance of the longest 5% of edges and PC1 is not statistically significant (*r* = 0.18 and *p* = 0.07). This small correlation could be partly, although not solely, because only a single hemisphere was considered, hence there are fewer long-distance connections. Note that other two parameter models, for example the economical preferential attachment generative model also discussed by Vértes et al., are not able to reproduce the global topology of the fMRI brain networks. Vértes et al. (2012) showed that this was the case in their dataset, based on the energy function that gave higher values for the preferential attachment model than the clustering model, indicating a worse fit to the real data. The same result holds in our dataset. Moreover, the preferential attachment model is unable to reproduce the motif fingerprint of the real fMRI brain networks, as demonstrated in section 10.2 of the Supplementary Information (Morgan et al., 2018). For a discussion of the ability of other network similarity measures to distinguish between the two models, see section 10.3 of the Supplementary Information (Morgan et al., 2018). Overall, these results show that the motif fingerprints are a powerful approach to capturing network topology, which is expected to be important for the function of complex networks.

**Figure 5.**
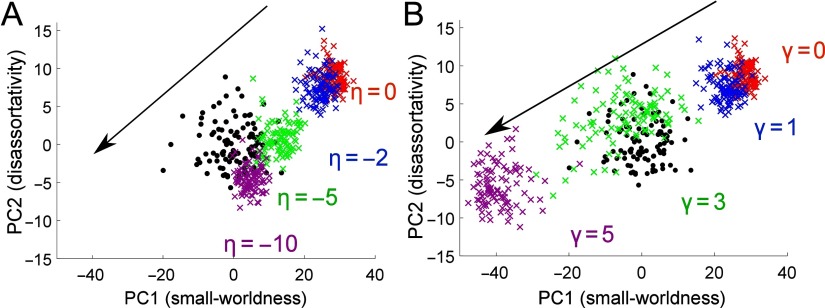
Generated networks with different *η* and *γ*. (A) Generated networks with different *η* and *γ* = 0 projected onto the space of the original networks. (B) Generated networks with different *γ* and *η* = 0 projected onto the space of the original networks. In both parts the original networks are shown as black dots and the superimposed networks are shown as colored crosses. Arrows denote the direction of increasing |*η*| or *γ*.

An advantage of the morphospace framework is that it allows us to explore the effect of changing the generative model parameters and to study how the factors of the model relate to one another. Therefore, lastly we generate networks with different *η*, setting *γ* = 0 and vice versa, and project the results onto the morphospace of our original fMRI brain networks. Note that the axes of the morphospace are still defined by the real fMRI brain networks. The results are shown in Figure 5. We note that changing either *η* or *γ* can change the networks’ positions along both PC1 and PC2, suggesting that both the distance and clustering penalizations can influence the networks’ motif fingerprints in similar ways. The heat maps of global network properties with different *η* and *γ* values shown in Supplementary Figure 13 also show that the distance and clustering parameters can compensate for each other to some extent.

## DISCUSSION

Our results show that, surprisingly, 97% of the variability in the motif fingerprints of fMRI brain networks can be explained by just two PCs. The fact that these data-driven motif PCs correlate significantly with several global metrics, including global efficiency, transitivity, and assortativity, is nontrivial and suggests that the motifs are able to capture key features of the networks’ topology. While we do not claim that the network motifs contain additional information to the wide range of global topological metrics that exist, using the motif PCs enables us to map the networks’ topology more comprehensively than any individual network measure without needing to select which network metrics to study a priori. The morphospace also enables us to explore the extent to which different metrics overlap and the relationships between them. Crucially, the space we obtain is low-dimensional, suggesting that the global topology of fMRI brain connectivity networks can be explained by few parameters.

The PCs of the morphospace represent the two main degrees of variability in the network motifs. The first PC (which alone explains over 80% of the variability) is negatively correlated with transitivity and positively correlated with global efficiency and small-worldness. There is also some preliminary evidence that the average length of the longest 5% of edges is positively correlated with PC1, although this result was not reproduced in the independent dataset and needs to be checked in other large datasets. Hence the main axis of variability in the structure of healthy fMRI networks appears to represent the difference between networks with high numbers of long-distance edges and high global efficiency, versus those with fewer long-distance edges, low global efficiency, and high transitivity, in line with many reports in the literature that small-worldness is a key factor in fMRI brain networks and encodes a trade-off between the topological benefits of long-distance edges and their cost (Bassett & Bullmore, 2006, 2016; Stam & van Straaten, 2012). PC2 is more novel and correlates most strongly with the assortativity of the networks. Assortativity has been linked to network robustness in the literature (Newman, 2002), however further work is needed to understand the exact role of assortativity in fMRI brain networks.

Generative models allow us to shed further light on the driving forces behind the two PCs. The motif fingerprints of the networks and their motif biplot can be reproduced using the two-parameter economical clustering generative model proposed by Vértes et al. (2012). Other generative models—including the economical preferential attachment model— are unable to reproduce the motif morphospace and hence the topology of the brain networks. This suggests that the economical clustering model is closer to the underlying biological mechanism than the economical preferential attachment model. This observation also demonstrates that it is not trivial to obtain a two-parameter model that can reproduce the networks (despite optimizing the model parameters) and shows that the motif morphospace provides a powerful way to distinguish between different generative models. Note that unlike in Vértes et al. (2012), in our work the key axes of variability in the topological properties are defined in a data-driven manner, using network motifs, giving us more confidence that the generative models capture the main axes of variability in the data, rather than a limited number of global metrics. Hence the result is a further test of the economical clustering model. In the future, we propose that models trying to reproduce fMRI brain networks use this motif morphospace approach to test their validity and to compare different models.

Varying the parameters *η* or *γ* in the generative model and projecting the resulting networks into the real fMRI brain network morphospace changes the networks’ motif fingerprints and their positions along both PC1 and PC2. This suggests that both *η* and *γ* are important in determining the networks’ motif fingerprints and positions along both PC1 and PC2 of the morphospace. Note that it is important to distinguish between (a) the variation in the positions of networks that all have a set value of *η* and *γ*, and (b) the variation in the positions of superimposed networks with different *η* and *γ* parameter values.

The former is due to random variations in the edges that are created (*η* and *γ* only set the probability of creating edges), while the latter is due to the changes in *η* and *γ*. One interesting question is what causes the topological differences in healthy fMRI brain networks described by the two PCs in our motif morphospace. Our results show that we can model a large amount of the variability in healthy fMRI networks with random fluctuations in a generative model. This suggests that the healthy population all follow the same underlying trade-offs (the parameters in the generative models), with fluctuations in how those trade-offs are navigated. For example, healthy brains make the same small-worldness trade-off between the topological benefits of long-distance edges and their cost, with fluctuations in the exact compromise reached. Unlike in healthy subjects, Vértes et al. (2012) suggested that in diseased states such as schizophrenia the underlying generative rules themselves are altered.

Further work is required to clarify the microscopic basis underlying these generative rules and their relationship with brain function, and for now we can only speculate as to what the mechanism might be. One possibility is that connections are not specifically established in a homophilic fashion but the homophilic connections are selected for and therefore get strengthened by Hebbian processes as other connections grow weaker. Indeed neuronal groups that share common inputs from the same topologically neighboring group are more likely to be simultaneously activated and therefore to consolidate direct connections. Another possibility is that there is a specific biological mechanism that implements a homophilic growth rule such as, for example, similar brain regions sharing similar time windows of development during neurogenesis. Interestingly, it was recently shown that cortical regions with similar cytoarchitecture are more likely to connect to one another than to regions with more different laminar structures (Goulas, Uylings, & Hilgetag, 2017). The authors suggested developmental time windows as a possible mechanism for this anatomical homophily. Finally, as mentioned in Vértes et al. (2012), enhanced probability of connection between neurons that already share nearest neighbors has been observed in the rat somatosensory cortex and was speculated to be genetically driven (Perin, Berger, & Markram, 2011). Ultimately, we note that is conceptually easier to imagine how homophily might be implemented biologically rather than for example preferential attachment or other rules that require each component of a brain network to have global information about the rest of the system.

Overall, the motif morphospace provides a data-driven, simple way to study and visualize network topology in a low-dimensional space. Our results suggest that this approach could also be useful in characterizing the topologies of other types of networks. Extending the approach to directed networks is straightforward. We note that in principle a motif morphospace could also be used to compare different types of networks.

### Limitations of the Study

FMRI brain networks enable functional connections in the brain to be mapped and have already been shown to be a powerful approach to studying the brain, proving able to distinguish patients’ brains from controls, for example (Arbabshirani, Plis, Sui, & Calhoun, 2017). However, the topology of fMRI brain networks is known to be biased by the fact that the networks are derived from correlating time series (or wavelet coefficients in our case; Zalesky et al., 2012). In order to show that our results are not solely driven by the topological bias inherent in estimating correlations, in Figure 1B we compare our motif fingerprint to the motif fingerprint of networks obtained from a null model proposed by Zalesky et al. (2012), in which the wavelet coefficients have been scrambled before being correlated with each other. We observe that the fingerprints are substantially different (none of the motif fingerprint scores are within one standard deviation of the real fMRI results). However, the fact that the networks are derived from correlations will still impact our results, because it affects the networks’ topology and the motifs capture that topology. In many ways this is a strength of the approach. More generally, which null model is most appropriate for fMRI brain networks is still a current topic of research. We believe that the motif morphospace approach presented in this paper will provide a useful method to study these questions in the future, because of the power of the motifs to map the topology of networks along their key axes of variability. The approach will also be useful in studying other types of brain networks, for example structural brain networks derived from diffusion tensor imaging.

Another limitation of the approach is that in order to compare networks’ motif fingerprints directly, we thresholded and binarized the networks with equal edge densities. Our results are robust to changing this fixed density, as discussed previously and shown in the Supplementary Information (Morgan et al., 2018), although fixing the edge density will result in a different threshold for different individuals. The binarization also means that the absolute values of the edges’ strengths are no longer taken into account.

Finally, as discussed already, our result showing that the Euclidean distance of the longest edges in the networks correlates with PC1 was not reproducible in our smaller Cambridge dataset and needs to be checked in other larger datasets.

## METHODS

We use 100 fMRI brain scans of healthy individuals from the Human Connectome Project. These data were already used in Termenon, Jaillard, Delon-Martin, & Achard (2016) to assess the reproducibility of the graph metrics. In this context, we downloaded the resting-state fMRI dataset publicly released as part of the Human Connectome Project (HCP), WU-Minn Consortium (for detailed parameters see Smith et al., 2013). The functional images were acquired on a 3T Siemens Connectome Skyra MRI scanner with a 32-channel head coil, using a multiband gradient-echo EPI imaging sequence with the following parameters: 2 mm isotropic voxels, 72 axial slices, TR = 720 ms, TE = 33.1 ms, flip angle = 52, field of view = 208 × 180 mm^2^, matrix size = 104 × 90 and a multiband factor of 8. A total of 1,200 images was acquired for a scan duration of 14 min and 24 s. The data used from the HCP include a second dataset with the same 100 subjects scanned a second time on different days with identical acquisition parameters. This retest dataset allowed us to confirm and validate our results.

In order to extract brain connectivity graphs, the structural and functional data were preprocessed according to the pipeline described by (Glasser et al., 2013). Finally, the functional data were registered to the individual structural image and further to the MNI152 atlas space using the transforms applied to the structural image (see Termenon et al., 2016 for details). In the present study, each brain image is parcellated using the classical automated anatomical labeling (AAL; Tzourio-Mazoyer et al., 2002) composed of 90 regions. The results were also robust to using a different parcellation (the AICHA parcellation, with 384 regions), as shown in section 3 of the Supplementary Information (Morgan et al., 2018).

In each parcel, regional mean time series are estimated by averaging, at each time point, the fMRI voxel values weighted by the gray matter probability of these voxels. Correlation matrices with 90 × 90 elements are computed using wavelets (Achard, Salvador, Whitcher, Suckling, & Bullmore, 2006), and wavelet scale four (corresponding to frequency interval [0.043–0.087] Hz) is chosen for extracting the graphs by thresholding the correlation matrices. The correlation threshold is applied to each individual network so that all graphs have the same number of edges. The cost, that is the ratio between the number of edges in the graph and the total number of possible edges, is fixed to approximately 20% (800 edges) based on the reproducibility results obtained in Termenon et al. (2016). Indeed, it was shown that for 20% cost, the test-retest reproducibility is higher for the majority of graph metrics (in this work we also test other thresholds of 400 and 1,200 edges, as shown in the Supplementary Information (Morgan et al., 2018). Each subject brain connectivity network is then represented as a graph G = (V, E), where V is the number of vertices or parcels (90 in our study), and E is the number of edges (800). The mean (standard deviation) of the correlations after thresholding was 0.625 (0.11) with 800 edges, 0.707 (0.10) with 400 edges, and 0.562 (0.12) with 1,200 edges. Note that the networks are constructed first by using a minimum spanning tree (MST), in order to avoid the spurious effect of disconnected nodes in the networks. Indeed as shown in Alexander-Bloch et al. (2010), global efficiency is biased by the presence of disconnected nodes, and a solution is firstly to compute an MST to connect all the nodes using the minimum number of edges with highest absolute values of correlations, 89 here. Based on these minimal networks, values of absolute correlations of edges are sorted by decreasing order and added to reach the cost defined previously. Above we report the average of the smallest correlation of the edges that are added excluding the MST. For a cost of 400 edges, on average, the MST adds 4.8% of the total number of edges where the absolute value of correlations is less than the reported threshold. For 800 edges, it adds on average 2.4% and for 1,200 edges, it adds on average 1.6%.

We then turn to characterize the decomposition of each graph G into motifs. We use the software FANMOD (Wernicke & Rasche, 2006) and Orca (Hoc̆evar & Dems̆ar, 2014) to count the number of motifs in each of the 100 networks (other software can also be used, for example mfinder; Milo et al., 2002). Unless otherwise stated, we focus on the six possible four-node undirected induced motifs shown in Figure 1A, which we denote as *m*_*i*_, where *i* ∈ 1, 6. We count the number of each of these motifs within each network, *N*_*i*_, and then normalize the number of motif *i* with respect to the total number of motifs in the network, to obtain the motif fingerprint for each network: $p_{i}=\frac{N_{i}}{\sum_{i}N_{i}}$.

We then perform a principal component analysis on the motif fingerprints for all of the 100 HCP networks. Here we have 100 observations (the subjects) and six variables (the fingerprint scores for each of the six possible four-node undirected induced motifs). The PCA was performed in MATLAB. The results of the PCA are displayed using a biplot of the principal component coefficients. The axes of the biplot represent the principal components, the observations are shown as red dots, and the original variables are plotted in the PC space as vectors.

Three random network models were used. The first generated random networks that did not preserve the degree distribution, using the function “makerandCIJ_und” from the Brain Connectivity Toolbox (Rubinov & Sporns, 2010). The second rewired the edges while preserving the degree distribution of the networks, using the function “randmio_und connected” from the Brain Connectivity Toolbox (Rubinov & Sporns, 2010), with a rewiring parameter of 15. Note that increasing the rewiring parameter to 25 did not change the resulting motif fingerprint. In the third random model the wavelet coefficients were scrambled, as proposed by Zalesky et al. (2012), using the Matlab function “randperm” to obtain random permutations. For the latter two null models, 100 randomized networks were calculated for each of the 100 real fMRI networks (giving 10,000 networks in total).

The global efficiency, assortativity, modularity, and transitivity of the networks were calculated using the Brain Connectivity Toolbox (Rubinov & Sporns, 2010). Note that transitivity is calculated as the ratio of triangles to triplets in the network (sometimes known as clustering). Modularity is calculated as the maximized modularity Q obtained by Newman’s spectral community detection (Newman, 2006). The total Euclidean distance is a sum of the Euclidean distance of all edges in the thresholded network, where the Euclidean distance of the functional edge between two brain regions is approximated as the distance between the centroids of the brain regions.

The generative models we compare the brain networks to are generated using the economical clustering model, given by Equation 1. We explore the parameter space manually to determine the values of *η* and *γ* that optimize an energy function calculated from the global efficiency, mean clustering coefficient, modularity, and degree distribution of the real HCP fMRI brain networks (see the Supplementary Information, Morgan et al., 2018, for more details). Following Vértes et al. (2012), we generate the networks with both hemispheres, but only consider a single hemisphere in subsequent calculations since the links between hemispheres are expected to follow different patterns to those within a single hemisphere. We use a threshold of 400 edges, as discussed in the Results section, and include a small number of additional edges if necessary to ensure that the graphs are connected.

## ACKNOWLEDGMENTS

The authors are grateful to Prof. Uri Alon, Dr. Miri Adler, and Dr. Pablo Szekely for sharing code. The authors would also like to thank Max Shinn for helpful discussions. E.T.B. is employed half-time by the University of Cambridge and half-time by GlaxoSmithKline; he holds stock in GSK.

## AUTHOR CONTRIBUTIONS

Sarah E. Morgan: Conceptualization; Formal analysis; Software; Visualization; Writing – original draft; Writing – review & editing. Sophie Achard: Conceptualization; Data curation; Writing – original draft; Writing – review & editing. Maite Termenon: Data curation. Edward T. Bullmore: Conceptualization; Supervision; Writing – original draft; Writing – review & editing. Petra E. Vértes: Conceptualization; Supervision; Writing – original draft; Writing – review & editing.

## FUNDING INFORMATION

P.E.V. is supported by a Medical Research Council Bioinformatics Research Fellowship (grant number MR/K020706/1). M.T. was supported by a grant from the Rhône-Alpes Région, France. S.A. was partly funded by a grant from la Région Rhône-Alpes and a grant from AGIR-PEPS, Université Grenoble Alpes-CNRS. Data were provided by the Human Connectome Project, WU-Minn Consortium (principal investigators: David Van Essen and Kamil Ugurbil; 1U54MH091657) funded by the 16 NIH Institutes and Centers that support the NIH Blueprint for Neuroscience Research; and by the McDonnell Center for Systems Neuroscience at Washington University.

## TECHNICAL TERMS

Functional magnetic resonance imaging (fMRI): An imaging technique that estimates brain activity by measuring changes in blood oxygenation levels locally, across the brain.
Generative model: A simple algorithm designed to grow networks that reproduce key observed topological properties with few parameters.
Global efficiency: A measure of the network’s integration, inversely related to the average shortest path lengths between all pairs of nodes.
Principal component analysis: A dimension-reduction technique, which reduces large numbers of variables to a few components that capture as much variability as possible.
Morphospace: A concept from evolutionary biology used to map the space of morphological properties of a set of organisms or systems.
Network motif: A small subgraph of the network, which can provide information about local connectivity patterns.
Small-world network: A network with both high clustering and short path length compared with randomized networks.
Transitivity: The ratio of triangles to triplets in the network, closely related to clustering.
Assortativity: The tendency of a node to connect to other nodes with similar degree.
