# Supplementary material for: Low-dimensional morphospace of topological motifs in human fMRI brain networks
Source: Netw Neurosci. 2018 Jun 1;2(2):285–302. doi: 10.1162/netn_a_00038 (PMC6130546; doi:10.1162/netn_a_00038)
Supplement: Supplementary file 1 [file netn-02-285-s001.pdf]

Morgan, S.E., Achard, S., Termenon, M., Bullmore, E.T., & Vértes, P.E. (2018). Supplemental Information for "Low dimensional morphospace of topological motifs in human fMRI brain networks." Network Neuroscience, 2(2), 285-302. https://doi.org/10.1162/netn_a_00038

Supplementary Information for: Low dimensional morphospace of topological motifs in human fMRI brain networks

Sarah E. Morgan[1], Sophie Achard[2], Maite Termenon[2], Edward T. Bullmore[1], and Petra E. Vértes[1]

[1]Brain Mapping Unit, Cambridge, UK
[2]GIPSA-Lab, Grenoble, France

* Corresponding author:
sem91@cam.ac.uk

Contents

1 Cumulative variability plot 2
2 Changing the threshold 3
3 Changing the parcellation 6
4 Re-test dataset 8
5 Changing the dataset 10
6 5 node motifs 12
7 Small-worldness coefficient 14
8 Including global metrics in the PCA 14
9 Relationship with Euclidean distance 17
9.1 Robustness 17
9.2 Exploring the location of the long-distance edges in the brain 17
10 Generative models 19
10.1 Economical clustering model 19
10.2 Economical preferential attachment model 20
10.3 Network similarity measures 21

1 Cumulative variability plot

Supplementary Figure 1 plots the amount of variability in the motif scores explained by each of the six principal components. As stated in the main text, 86% of the motif variability is explained by the first principal component and 97% is explained by the first two principal components.

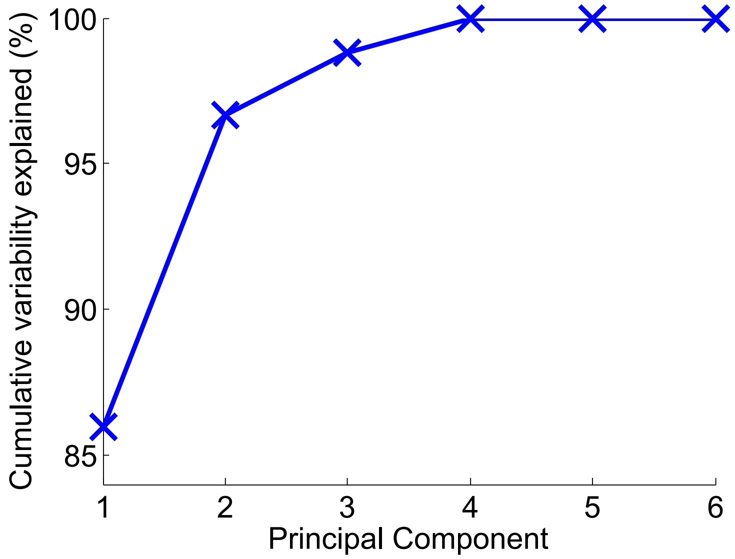

Supplementary Figure 1: Cumulative variability explained by each principal component (%)

2 Changing the threshold

The motif fingerprint and biplot in the main text are given for networks with 800 edges. Results with 400 edges and 1200 edges are similar, as is the cumulative variability plot, as shown in Supplementary Figures 2 and 3.

The correlations between PCs 1 and 2 and the global metrics and the average Euclidean distance of the longest 5% of edges are also similar in networks with 400 or 1200 edges, as shown in Supplementary Table 1. Here both the Pearson correlation coefficients (r) and the p-values for Pearson's correlation using a Student's t distribution are shown. Note that with 1200 edges, the direction of PC2 is reversed with respect to the underlying motifs, hence the sign of the correlations of global metrics with PC2 are also reversed. However, since this direction is arbitrary, the change in sign is not important.

		Global efficiency	Transitivity	Assortativity	Eucl. distance of longest 5% of edges
400 edges	PC1	0.74 (< 0.001)	-0.89 (< 0.001)	-0.62 (< 0.001)	0.37 (< 0.001)
	PC2	0.32 (0.001)	-0.36 (< 0.001)	-0.64 (< 0.001)	0.13 (0.21)
800 edges	PC1	0.82 (< 0.001)	-0.94 (< 0.001)	-0.36 (< 0.001)	0.50 (< 0.001)
	PC2	0.20 (0.04)	-0.28 (0.004)	-0.81 (< 0.001)	0.16 (0.11)
1200 edges	PC1	0.85 (< 0.001)	-0.96 (< 0.001)	-0.31 (0.002)	0.49 (< 0.001)
	PC2	0.00029 (0.998)	0.20 (0.05)	0.80 (< 0.001)	-0.20 (0.05)

Supplementary Table 1: The correlations between the PCs and the global metrics and the average Euclidean distance of the longest 5% of edges are also similar in networks with 400 or 1200 edges. The Pearson correlation coefficients are shown. The p-values for Pearson's correlation using a Student's t distribution are also shown in brackets.

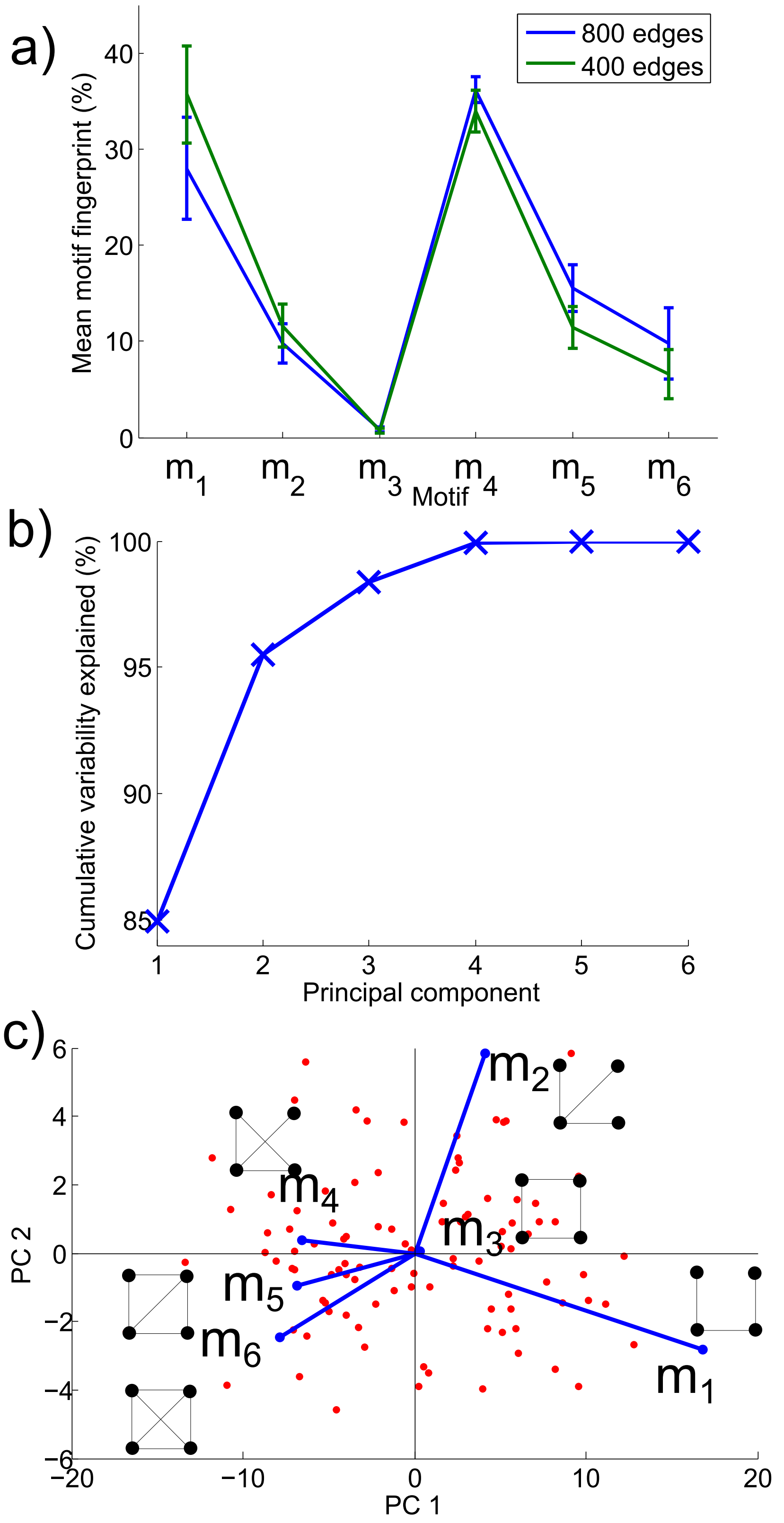

Supplementary Figure 2: a) Motif fingerprint, b) Cumulative variability and c) Motif biplot for networks with 400 edges. The motif fingerprint with 800 edges is also shown for comparison.

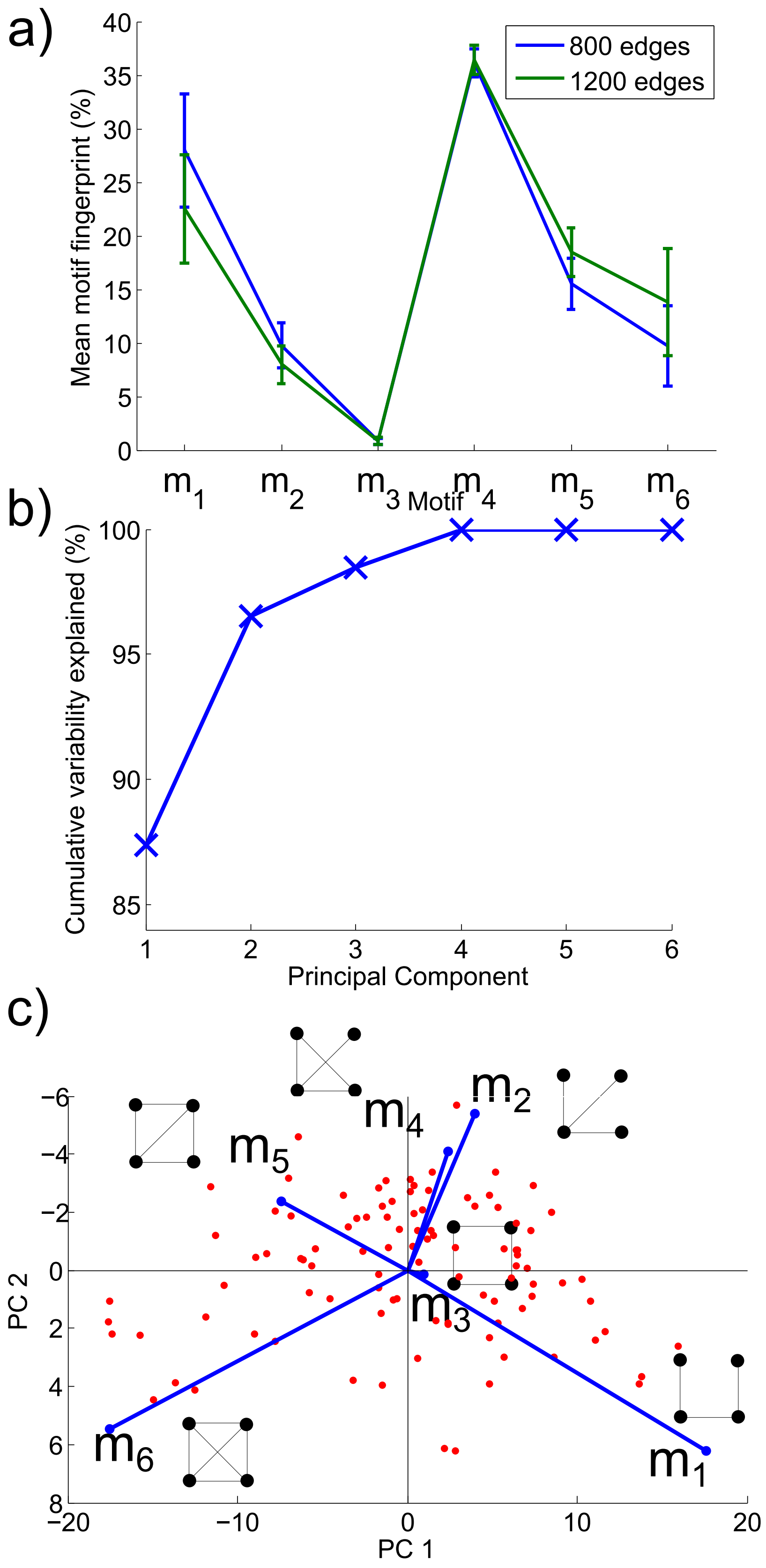

Supplementary Figure 3: a) Motif fingerprint, b) Cumulative variability and c) Motif biplot for networks with 1200 edges. The motif fingerprint with 800 edges is also shown for comparison.

3 Changing the parcellation

Throughout the main text, we use the AAL parcellation with 90 regions. Here we show that our results are robust to changing the parcellation. In particular, we consider the AICHA parcellation [1] with 384 regions. This parcellation aims to preserve homotopy. In our case, we were interested in testing our results using a parcellation with a larger number of regions to test the robustness of the motifs extraction.

As before, the networks are thresholded at approximately 20%, with 14689 edges per network in this case. The motif fingerprint and biplot we obtain are shown in Supplementary Figure 4 and are remarkably similar to those from our data and parcellation shown in Figure 1 of the main text. We note that an even higher percentage of variability is explained by the first PC (over 92% of the total variability). As shown in Supplementary Table 2, the correlations with global metrics and the average Euclidean distance of the longest 5% of edges are also very similar to our previous results- PC1 correlates positively with global efficiency and negatively with transitivity. PC2 correlates positively with assortativity- note that the change in sign with respect to our previous results is explained by the fact that PC2 is 'flipped' with respect to the underlying motifs. This change in sign is unimportant because the direction of PC2 is arbitrary.

	Global efficiency	Transitivity	Assortativity	Euclidean distance of longest 5% of edges
PC1	0.94 (< 0.001)	-0.98 (< 0.001)	-0.27 (0.006)	0.53 (< 0.001)
PC2	0.11 (0.26)	0.13 (0.20)	0.58 (< 0.001)	-0.26 (0.01)

Supplementary Table 2: The correlations between the PCs and the global metrics and the average Euclidean distance of the longest 5% of edges, using the HCP data with the AICHA parcellation. All results are shown for networks with 14689 edges, which is an approximately 20% threshold.

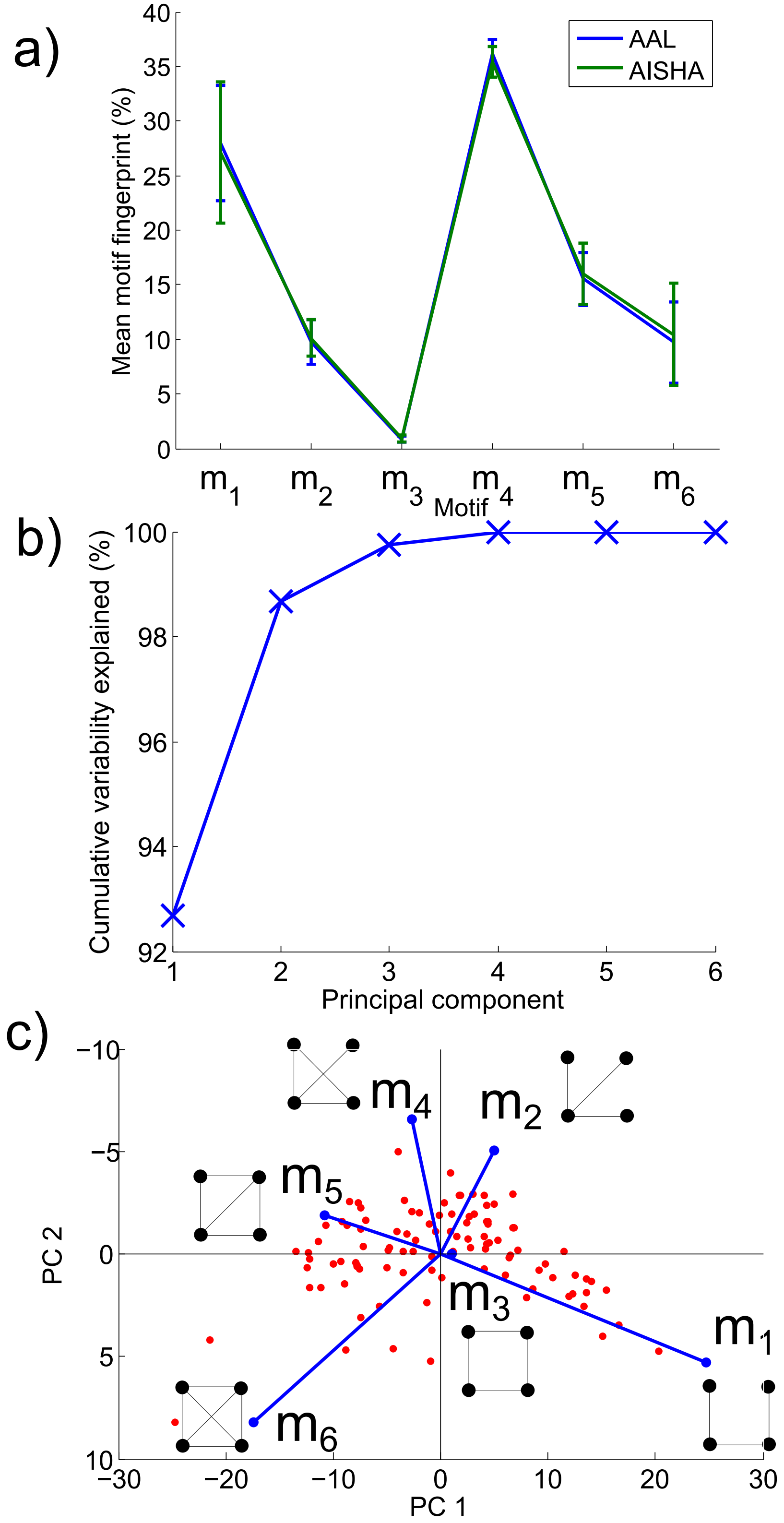

Supplementary Figure 4: a) Motif fingerprint, b) Cumulative variability and c) Motif biplot for HCP dataset with the AICHA parcellation. The motif fingerprint in the AAL parcellation is also shown for comparison.

4 Re-test dataset

Using a re-test HCP dataset, in which all 100 subjects were scanned a second time with identical acquisition parameters, we obtain a similar motif fingerprint, cumulative variability plot and motif biplot as before. Results are shown in Supplementary Figure 5. The correlations with global metrics and with the average Euclidean distance of the longest 5% of edges are also similar in the re-test HCP dataset, see Supplementary Table 3. All results are shown for networks with 800 edges, as in the main text.

	Global efficiency	Transitivity	Assortativity	Euclidean distance of longest 5% of edges
PC1	0.82 (< 0.001)	-0.90 (< 0.001)	-0.41 (< 0.001)	0.43 (< 0.001)
PC2	0.25 (0.01)	-0.38 (< 0.001)	-0.71 (< 0.001)	0.02 (0.82)

Supplementary Table 3: The correlations between the PCs and the global metrics and the average Euclidean distance of the longest 5% of edges, using the re-test HCP data. All results are shown for networks with 800 edges in the AAL 90 parcellation, which is an approximately 20% threshold.

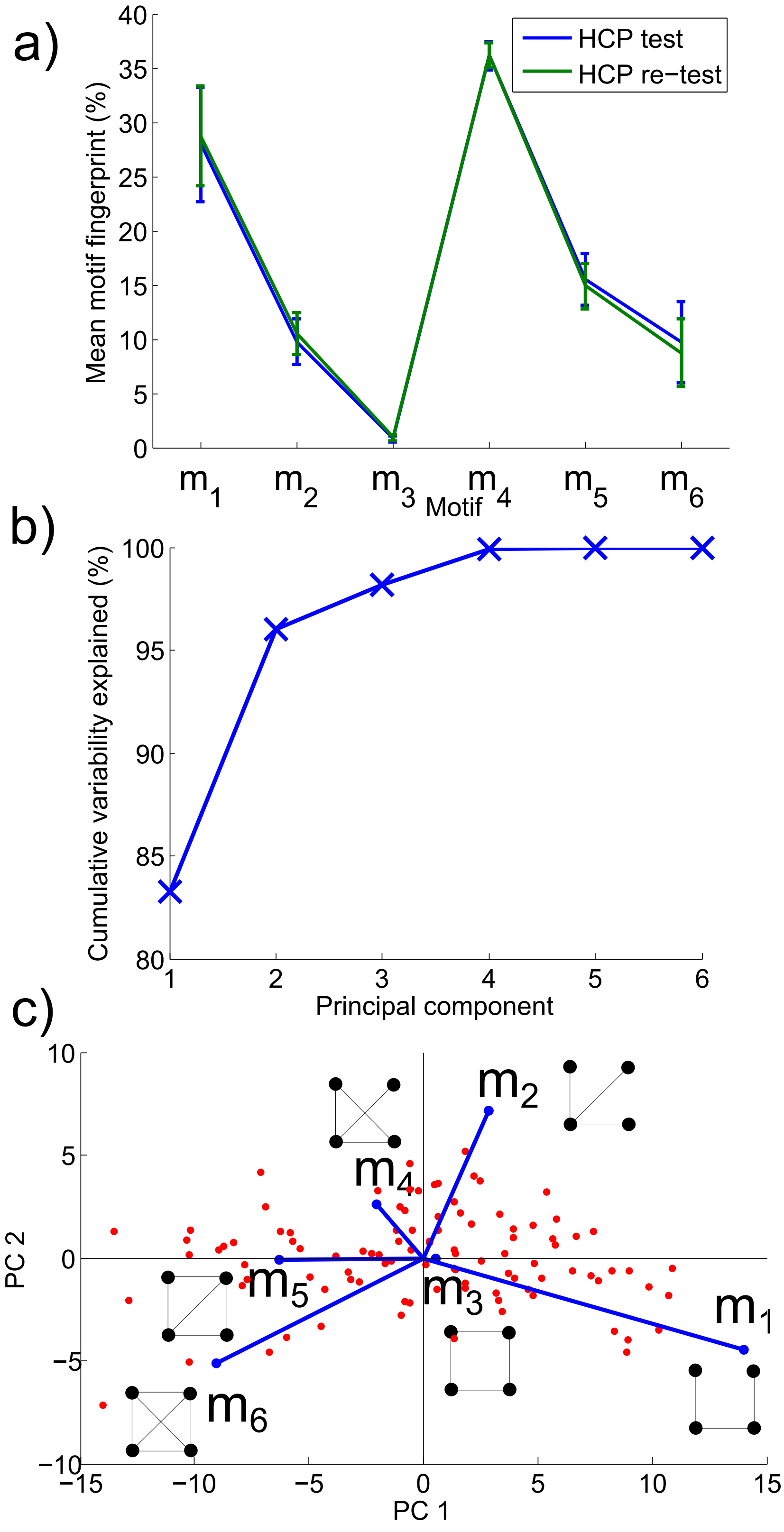

Supplementary Figure 5: a) Motif fingerprint, b) Cumulative variability and c) Motif biplot for re-test dataset. All parts have networks with 800 edges.

5 Changing the dataset

Above we showed that our results are robust in the HCP re-test dataset. We now test them in an independent dataset, which we refer to as the 'Cambridge' dataset. This dataset has been reported previously in [2] and includes 26 subjects of varying ages. Resting-state fMRI data was acquired using classical Gradient-echo planar imaging (EPI) in the Wolfson Brain Imaging Center (Cambridge, UK) (see [1] for the details of the parameter acquisition). The length of the acquisition was 9.6 minutes with a repetition time equal to 1.1 s. The same pre-processing procedure was applied as before to obtain time series with 90 anatomical regions and 512 samples in time. Again as before, the AAL parcellation was used and the networks are thresholded at 800% edges (approximately 20% cost).

The motif fingerprint and biplot we obtain are shown in Supplementary Figure 6 and are again similar to those from the HCP dataset shown in Figure 1 of the main text. The correlations with global metrics are very similar to our previous results, as shown in Supplementary Table 4, PC1 correlates positively with global efficiency and negatively with transitivity, whilst PC2 correlates negatively with assortativity.

The only result which was significant in the HCP dataset and is no longer significant in this dataset is the correlation between the average Euclidean distance of the longest 5% of edges. Whilst we still find a positive correlation in the Cambridge dataset (r=0.30), the p-value is > 0.05 (p=0.14). The reason for this difference is unclear, but could be due to the smaller number of subjects available in the Cambridge dataset. Further work is needed to clarify the reason for this difference.

	Global efficiency	Transitivity	Assortativity	Euclidean distance of longest 5% of edges
PC1	0.87 (< 0.001)	-0.88 (< 0.001)	0.18 (0.37)	0.30 (0.14)
PC2	0.34 (0.09)	-0.45 (0.02)	-0.84 (< 0.001)	0.16 (0.44)

Supplementary Table 4: The correlations between the PCs and the global metrics and the average Euclidean distance of the longest 5% of edges in the Cambridge dataset. All results are shown for networks with 800 edges.

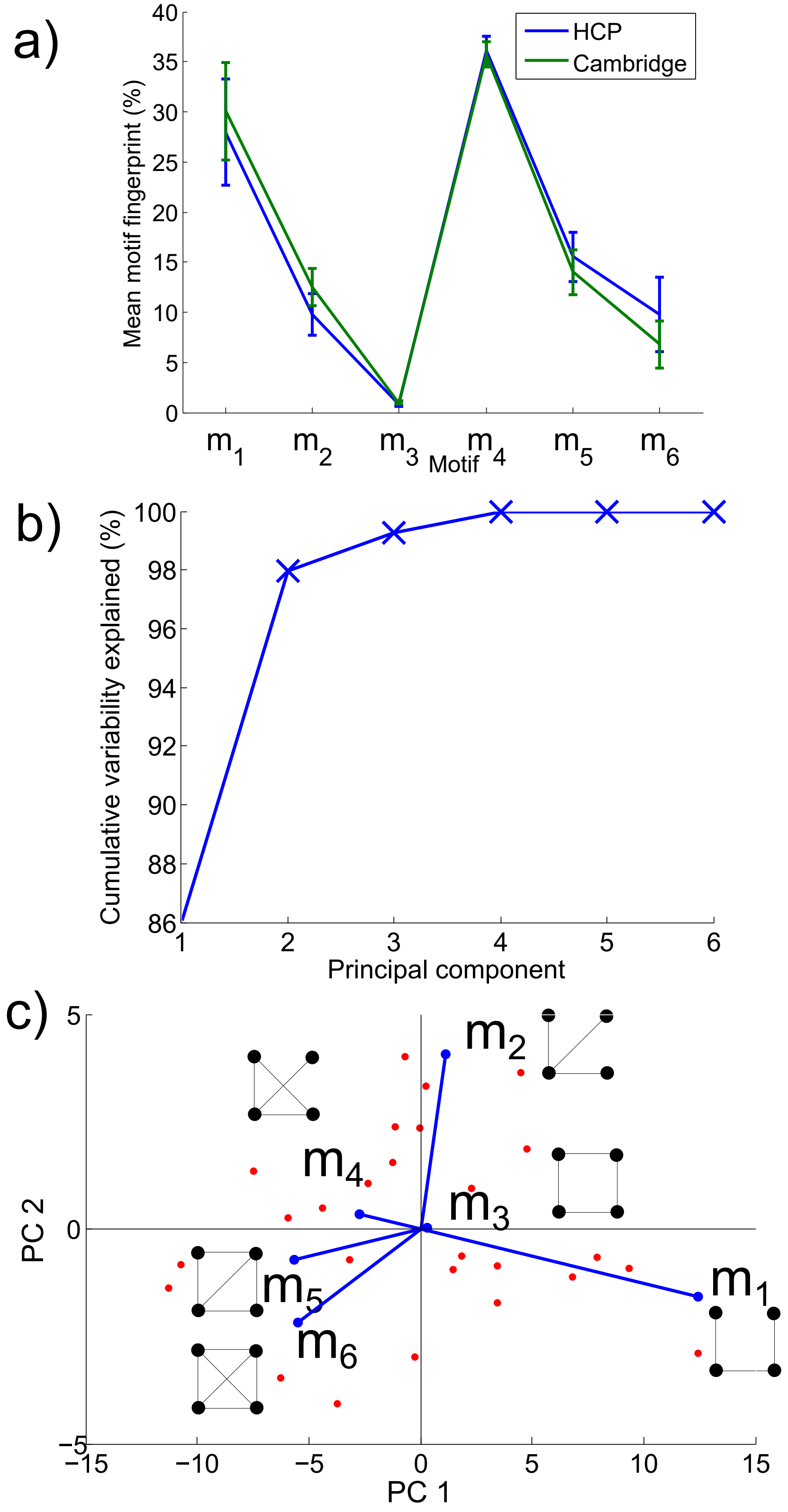

Supplementary Figure 6: a) Motif fingerprint, b) Cumulative variability and c) Motif biplot for the Cambridge dataset.

6 5 node motifs

In Figure 1 of the main text, we present the cumulative variability and motif biplot for 4 node motifs. In Supplementary Figure 7, we show the same plots for 5 node motifs. Again, the first two principal components explain over 90% of the motif variability, even though there are 21 possible 5 node motifs. Hence there is significant overlap between the information contained within the motif counts. We note that PC1 explains slightly less of the variability than it did in the 4 node motif case and PC2 explains slightly more (74% instead of 86% and 18% instead of 11%). Figure 7 b plots the motif biplot for 5 node motifs, which is similar to the 4 node biplot; highly clustered motifs have low PC1, whilst 'chain-like' motifs have high PC1. PC2 reflects the assortativity of the motifs. The correlations with global metrics are shown in Supplementary Table 5 and are remarkably similar to those obtained with 4 node motifs.

	Global efficiency	Transitivity	Assortativity	Euclidean distance of longest 5% of edges
PC1	0.80 (< 0.001)	-0.88 (< 0.001)	-0.24 (0.01)	0.49 (< 0.001)
PC2	0.35 (< 0.001)	-0.43 (< 0.001)	-0.79 (< 0.001)	0.19 (0.05)

Supplementary Table 5: The correlations between the PCs and the global metrics and the average Euclidean distance of the longest 5% of edges in the HCP data with 5 node motifs. All results are shown for networks with 800 edges.

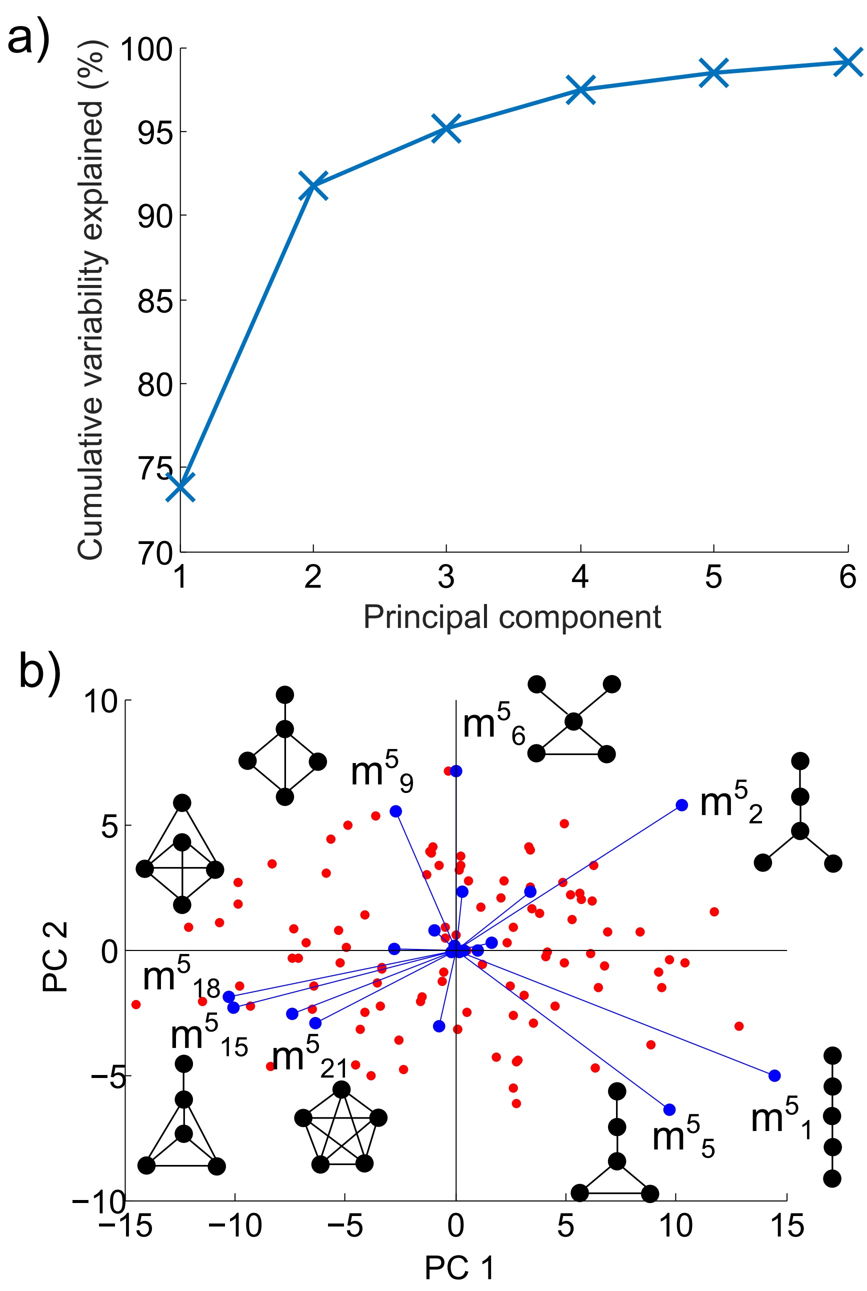

Supplementary Figure 7: a) Cumulative variability of the 5 node motif fingerprints. b) 5 node motif biplot for 100 HCP fMRI brain networks, with key motifs labelled. In both parts, the networks were thresholded with 800 edges, as in Figure 1 of the main text.

7 Small-worldness coefficient

The small-worldness coefficient [3] correlates with PC1, with r=0.87 ($p < 0.001$). There is no significant correlation with PC2 (r=-0.15, p=0.12). The motif morphospace coloured by small-worldness coefficient is shown in Supplementary Figure 8.

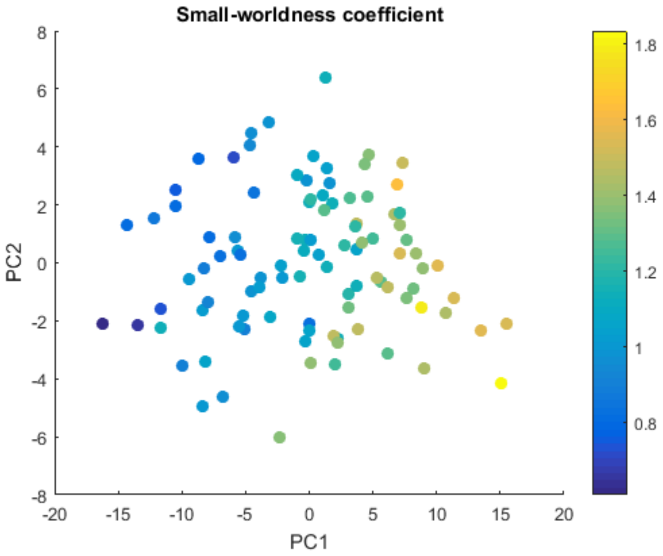

Supplementary Figure 8: Motif morphospace coloured according to the networks' small-worldness coefficients.

8 Including global metrics in the PCA

Supplementary Figures 9 b and d plot the cumulative variability plot and the biplot with efficiency, transitivity and assortativity included in the PCA in addition to the motif scores. Note that in order to be able to compare the motif scores to the global metrics in the PCA we have first taken z-scores of each of the nine inputs (the six motif scores and the three global metrics). The cumulative variability plot and the biplot with only the six z-scored motif scores are shown in Supplementary Figures 9 a and c for comparison. Supplementary Tables 6 and 7 provide the loadings from the PCA with and without the global metrics included.

	PC1	PC2
m_1	0.37	-0.24
m_2	0.29	0.42
m_3	0.30	-0.34
m_4	-0.17	0.57
m_5	-0.39	0.02
m_6	-0.40	-0.08
Efficiency	0.37	0.02
Transitivity	-0.41	-0.04
Assortativity	-0.21	-0.56

Supplementary Table 6: Loadings for PCA with efficiency, transitivity and assortativity included.

As can be seen from the biplots, the motif directions are similar in both cases (the sign of PC1 is flipped, but this difference is unimportant since the sign is arbitrary). According to the sign convention in the original

	PC1	PC2
m_1	-0.48	-0.13
m_2	-0.29	0.61
m_3	-0.41	-0.32
m_4	0.28	0.65
m_5	0.47	-0.15
m_6	0.47	-0.26

Supplementary Table 7: Loadings for PCA without global metrics (having calculated z-scores for the motif scores, see text for details).

plot, efficiency is positively weighted along PC1, whilst transitivity is negatively weighted. Assortativity is negatively weighted along PC2 as well as PC1. These results are in line with the correlations between the global metrics and the PCs shown previously. From Supplementary Table 6, when the global metrics are included in the PCA, the largest loadings for PC1 are for transitivity (-0.41) and m_6 (-0.40), whilst the largest loadings for PC2 are for m_4 (0.57) and assortativity (0.56). Hence the global metrics and the motif scores show similar loadings.

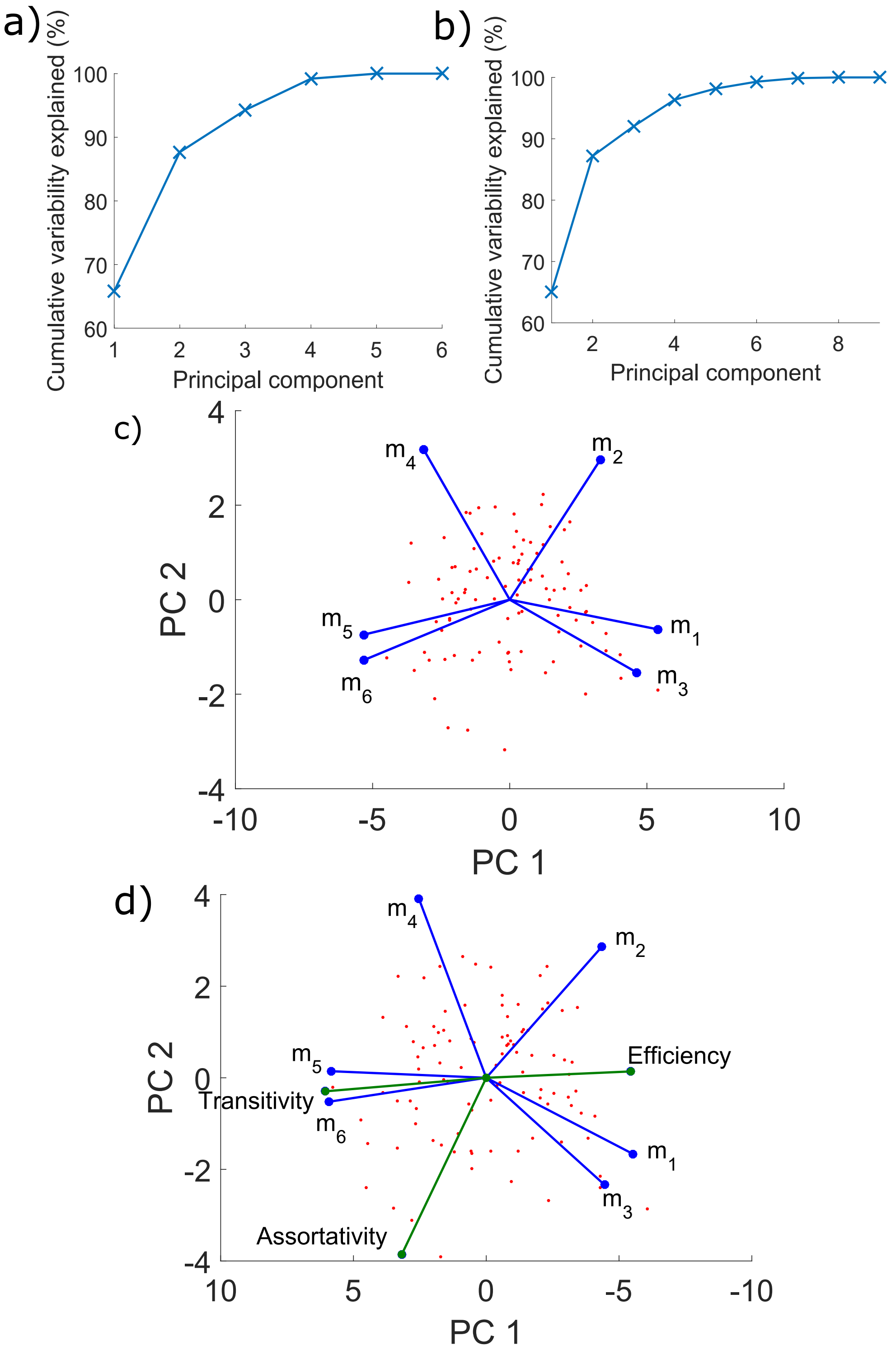

Supplementary Figure 9: Cumulative variability plots for a) the PCA calculated from 6 motif z-scores per network and b) the PCA calculated from 6 motif z-scores in addition to global efficiency, transitivity and assortativity for each network. c) and d) plot the corresponding biplots.

9 Relationship with Euclidean distance

9.1 Robustness

In the main text, we reported the correlation of the average Euclidean distance of the longest 5% of edges with PC1 and PC2, for the HCP networks. The choice of 5% was somewhat arbitrary, however similar results can be obtained with the longest 10% or 1% of edges, as shown in Table 8.

	10%	5%	1%
PC1	0.42 (< 0.001)	0.50 (< 0.001)	0.57 (< 0.001)
PC2	0.22 (0.03)	0.16 (0.11)	0.05 (0.61)

Supplementary Table 8: The correlations between PC1 and PC2 and the average Euclidean distance of the longest 10%, 5% and 1% of edges in our networks.

9.2 Exploring the location of the long-distance edges in the brain

In the main text we compare the distributions of the Euclidean distances of the edges for two example subjects (Figure 1, parts c and d). In Supplementary Figures 10 and 11 we show the longest 5% of edges from these two subjects plotted on the brain. We observe that the long distance edges in the subject represented by a circle (whose longest edges are shorter on average) are more likely to be interhemispheric, whilst the subject represented by a square (whose longest edges are longer on average) are more likely to be intrahemispheric, often connecting the front and the back of the brain. Note that this does not mean that the subject denoted by the circle has more interhemispheric edges in total than the subject denoted by the square, rather that more of their longest edges are interhemispheric.

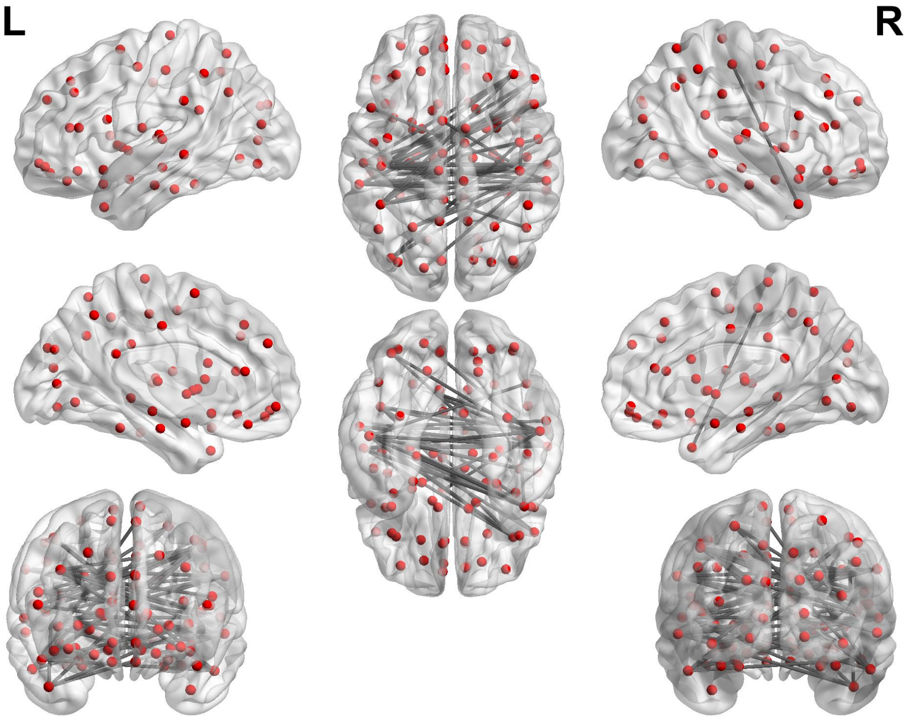

Supplementary Figure 10: Longest 5% of edges for the subject represented by the circle in Figure 2 of the main text, visualised with the BrainNet Viewer [4].

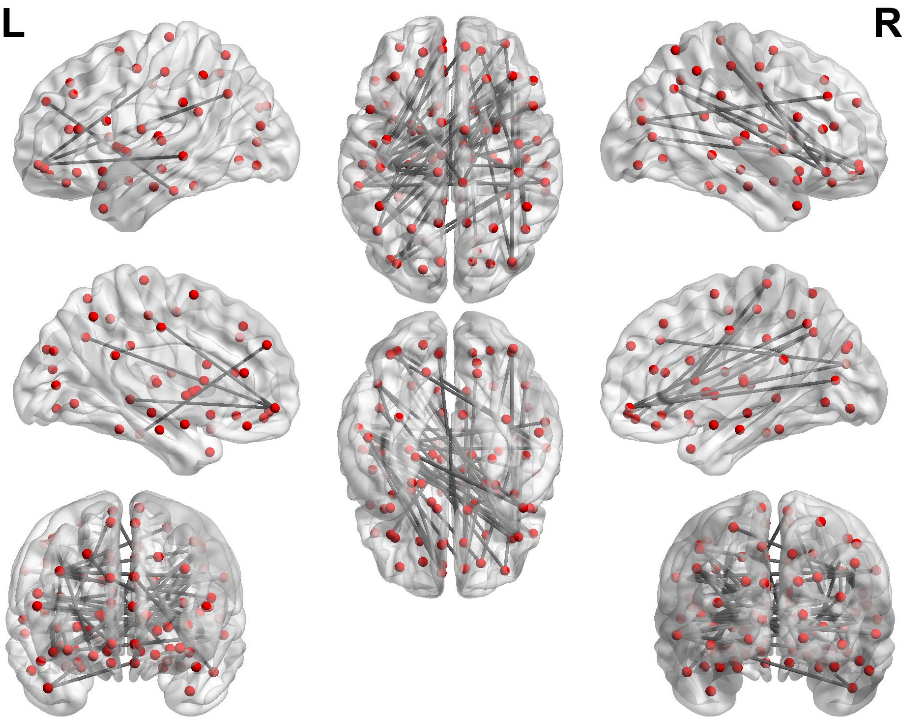

Supplementary Figure 11: Longest 5% of edges for the subject represented by the square in Figure 2 of the main text, visualised with the BrainNet Viewer [4].

To explore this finding further in the whole dataset, we counted the number of the longest 5% of edges in each subject which were interhemispheric. Note that 5% of the edges in the network corresponds to 40 edges in our networks (since they have 800 edges in total), hence the largest number of these edges which can be interhemispheric is 40. In other words, for each network we listed its 800 edges by the Euclidean distance between the two regions they connect, selected the 40 longest edges and then counted how many of these edges were interhemispheric. Supplementary Figure 12 plots the motif morphospace coloured by the number of the longest 5% of edges in each subject which were interhemispheric. As expected from Supplementary Figures 10 and 11, we observe that the results correlate with PC1 ($r=-0.44$, $p< 0.001$), in other words the longest edges in networks with high PC1 scores (which tend to have high global efficiency) are more likely to be long distance edges within a single hemisphere than the longest edges in networks with low PC1 scores, which are more likely to be interhemispheric. Importantly, there is no correlation between PC1 or PC2 and the total number of interhemispheric edges in the network, hence on average networks with low or high PC1 scores still have the same number of interhemispheric edges in total.

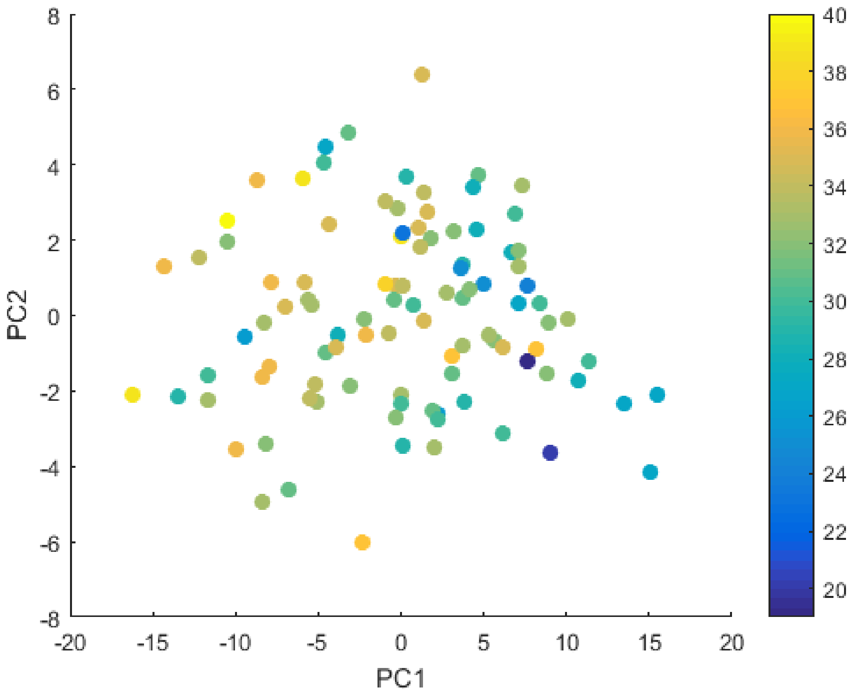

Supplementary Figure 12: Motif morphospace coloured according to the number of the longest 5% of edges in each subject which are interhemispheric.

10 Generative models

10.1 Economical clustering model

In order to determine η and γ for the generative model, we generated networks with a wide range of η and γ values and calculated the goodness of fit of the generated networks to the HCP brain networks. The goodness of fit was determined using the same energy function used by Vertes et al. [5], which optimises the networks for modularity, mean local clustering efficient, global efficiency and degree distribution. Note that the networks were not optimised for the motif fingerprint, or assortativity. Supplementary Figure 13 plots the energy function as a function of η and γ. The lower the energy, the better the fit to the networks. The minimum is found at $\gamma = 3$ and $\eta = -2.5$, therefore in the main text we set these values for η and γ. Note that as η and γ increase, the networks become more clustered, as can be seen from Figure 5 in the main text and the motif distribution shown in Supplementary Figure 14. Therefore increasing η and γ further is not expected to give a better approximation to the HCP brain networks.

The correlations between the PCs and the global efficiency, assortativity, transitivity and the average length of the longest 5% of edges are shown in Table 9. The results for global efficiency, transitivity and assortativity are similar to those observed in the real data with a single hemisphere only and a 10% threshold. The correlation between the Euclidean distance of the longest 5% of edges and PC1 is not statistically significant in the generated networks (p=0.07). This is partly, although not solely, due to the reduced correlation between the Euclidean distance of the longest 5% of edges and PC1 when only a single hemisphere is considered, which is unsurprising because there are fewer long distance connections. We also note that with a single hemisphere only, the global efficiency of both the real networks and the generated networks shows strong correlation with PC2 as well as with PC1. Interhemispheric connections are known to play an important role in the brain and further work is needed to fully understand their influence on the motif morphospace.

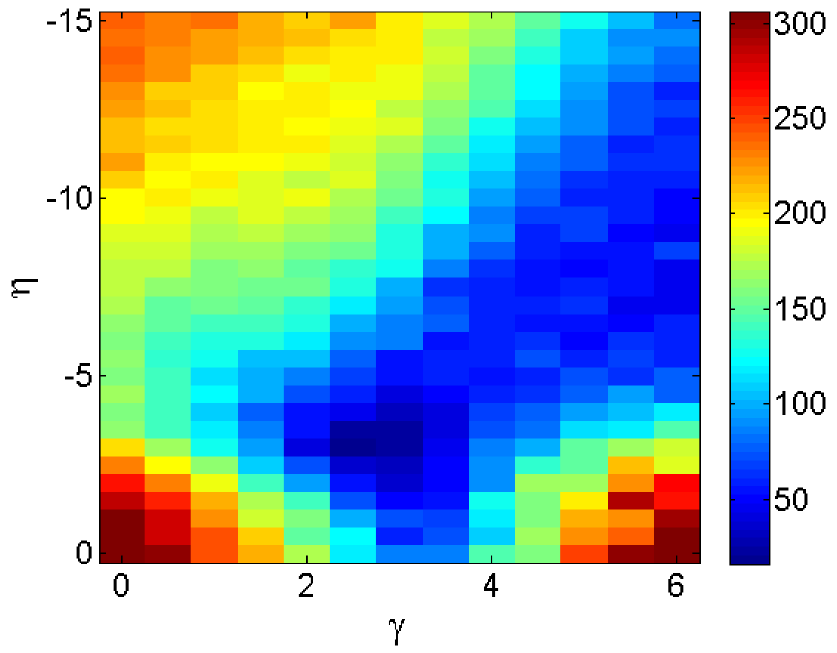

Supplementary Figure 13: The average energy values of networks generated using the economical clustering model as η and γ are varied (mean of 100 generated networks). The energy values are shown as $\log_{10}(E)$, where E is as defined in [5].

		Global efficiency	Transitivity	Assortativity	Eucl. distance of longest 5% of edges
Generated	PC1	0.49 ($<$ 0.001)	-0.80 ($<$ 0.001)	-0.41 ($<$ 0.001)	0.18 (0.07)
	PC2	0.53 ($<$ 0.001)	-0.50 ($<$ 0.001)	-0.74 ($<$ 0.001)	0.07 (0.50)
HCP	PC1	0.57 ($<$ 0.001)	-0.76 ($<$ 0.001)	-0.51 ($<$ 0.001)	0.27 (0.007)
	PC2	0.58 ($<$ 0.001)	-0.44 ($<$ 0.001)	-0.68 ($<$ 0.001)	-0.003 (0.97)

Supplementary Table 9: The correlations between the PCs and the global metrics and the average Euclidean distance of the longest 5% of edges in generated and real fMRI brain networks with a 10% threshold and a single hemisphere. The Pearson correlation coefficients are shown. The p-values for Pearson's correlation using a Student's t distribution are also shown in brackets.

10.2 Economical preferential attachment model

Whilst the economical clustering model is able to reproduce the motif fingerprints from the real data, other models are not. For example, here we show results from the economical preferential attachment model, also described in [5]. As above, in order to determine η and γ we explore the goodness of fit of networks across the parameter space manually. The energy at different η and γ is shown in Supplementary Figure 15. Note that the energy is always higher than the optimal energy values obtained from the economical clustering model, suggesting that networks generated by the economical preferential attachment model are not as close a fit to the real fMRI brain networks. The solutions with the lowest energy values form a band across the parameter space. Since it is difficult to ascertain whereabouts in the band the optimal solution is, in Supplementary Figure 16 we show motif fingerprints for a range of η and γ values within the lower energy band. None of the motif fingerprints are in agreement with the original networks. For $\gamma = 1$ and $\eta = -4$, the proportions of m_1 and m_2 are higher than in the real brain networks, whilst the proportion of m_4 is too low. As γ and $|\eta|$ increase, the proportion of m_1 decreases and the proportion of m_4 increases, however the proportion of m_2 increases away from the results for the real brain networks. This trend continues at higher values of η and γ. Hence the economical preferential attachment model is unable to reproduce the motif fingerprint, unlike

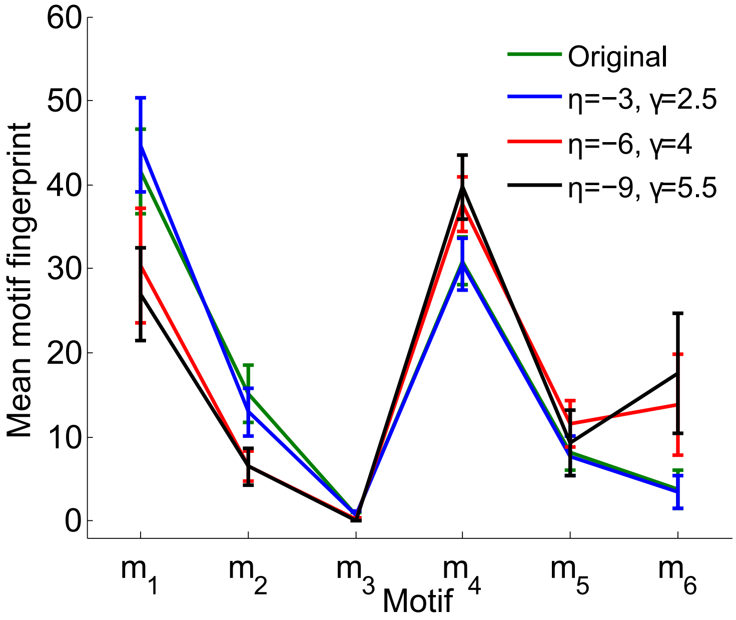

Supplementary Figure 14: Motif distributions of networks generated using the economical clustering model with different η and γ values. As γ increases and η becomes more negative, the networks become more clustered, hence the proportion of m_6 increases and the proportion of m_1 decreases.

the economical clustering model. This illustrates that it is not trivial to find a model which can reproduce the motif fingerprint.

10.3 Network similarity measures

An alternative way to compare the results from the generative models to the real fMRI networks is to use network similarity measures. In Supplementary Table 10 we use three network similarity measures to measure the differences between 1) the economical clustering model (with $\eta = -3$ and $\gamma = 2.5$) and the real fMRI networks, 2) the preferential attachment model (with $\eta = -20$ and $\gamma = 6$) and the real fMRI networks and 3) random networks with the same number of nodes and edges as the real fMRI networks (generated using the Brain Connectivity Toolbox function makerandCIJ_und.m [6]) and the real fMRI networks. In each case we average the measure over all possible pairwise comparisons, using the 100 real networks and 100 generated/random networks. We report the mean average and the standard deviation of the results. The three measures we use are: 1) the number of edges the networks have in common (out of a possible total of 99 edges), 2) the Hamming distance and 3) a recently reported network dissimilarity measure, using the default parameters described in the paper (w_1=w_2=0.45, $w_3 = 0.1$) [7]. Note that this measure has a range from zero to one and is equal to zero in the case of identical networks, hence lower numbers mean a higher similarity.

We find that the differences between the real fMRI networks and the economical clustering model or the preferential attachment model are within one standard deviation from each other with all three methods, unlike the differences between the real fMRI networks and the random networks, which are much more substantial. For example, the number of edges in common with the real fMRI networks is 24.7 ± 4.1 and 26.6 ± 3.8 for the economical clustering model and the preferential attachment model respectively, compared to only 9.9 ± 2.9 edges in common between the real fMRI networks and the random networks. Nonetheless, a two-sided Wilcoxon signed rank test shows that there are statistically significant differences between the similarity measures comparing the 100 real fMRI networks and the two generative models. In particular,

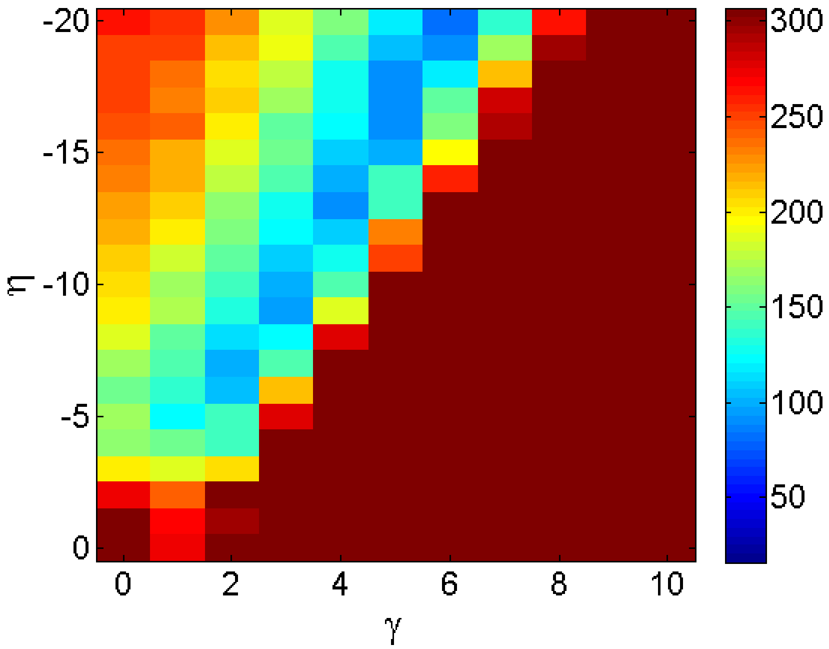

Supplementary Figure 15: The average energy values of networks generated using the economical preferential attachment model as η and γ are varied (mean of 100 generated networks). The energy values are shown as $\log_{10}(E)$, where E is as defined in [5].

the preferential attachment model appears to be closer to the real networks in terms of the number of edges in common and the Hamming distance, whilst the economical clustering model is closer using the method proposed by Schieber et al (which was designed to account for topological properties of the networks).

	No. of edges in common	Hamming distance	Schieber et al [7]
Economical clustering model vs fMRI	24.7 ± 4.1	303.8 ± 17.1	0.08 ± 0.04
Preferential attachment model vs fMRI	26.6 ± 3.8	291.3 ± 14.6	0.09 ± 0.04
Random networks vs fMRI	9.9 ± 2.9	356.4 ± 11.5	0.20 ± 0.04

Supplementary Table 10: Comparison of generated and real fMRI brain networks using three network similarity measures. See text for details.

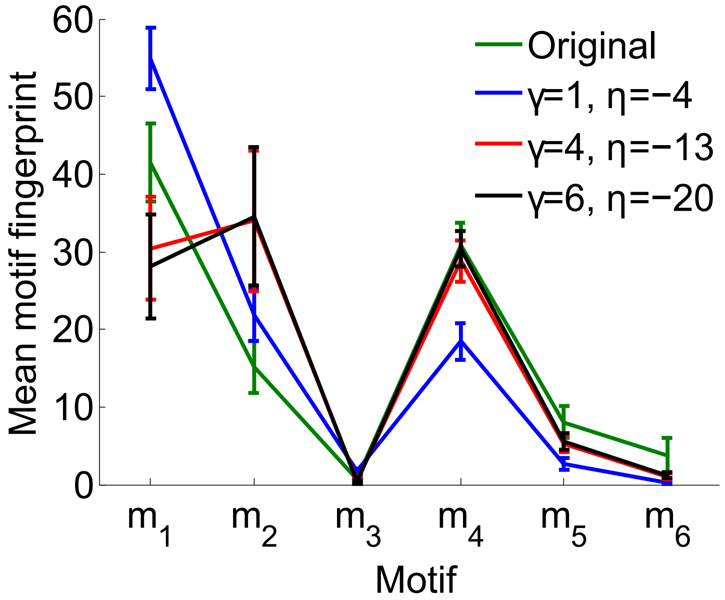

Supplementary Figure 16: Motif distributions of networks generated using the economical preferential attachment model with different η and γ values. The generated networks are unable to reproduce the real brain network's motif fingerprint.

References

[1] M. Joliot, G. Jobard, M. Naveau, N. Delcroix, L. Petit, L. Zago, F. Crivello, E. Mellet, B. Mazoyer, and N. Tzourio-Mazoyer, "Aicha: An atlas of intrinsic connectivity of homotopic areas," *J. Neurosci. Methods*, vol. 254, pp. 46–59, 2015.

[2] S. Achard and E. Bullmore, "Efficiency and cost of economical brain functional networks," *PLoS Computational Biology*, vol. 3(2), p. e17, 2007.

[3] M. Humphries, K. Gurney, and T. Prescott, "The brainstem reticular formation is a small-world, not scale-free, network," *Proc. Biol. Sci.*, vol. 273, pp. 503–511, 2006.

[4] M. Xia, J. Wang, and Y. He, "BrainNet Viewer: A network visualization tool for human brain connectomics," *PLoS ONE*, vol. 8, p. e68910, 2013.

[5] P. Vértes, A. Alexander-Bloch, N. Gogtay, J. Giedd, J. Rapoport, and E. Bullmore, "Simple models of human brain functional networks," *Proc. Natl. Acad. Sci.*, vol. 109, pp. 5868–73, 2012.

[6] M. Rubinov and O. Sporns, "Complex network measures of brain connectivity: Uses and interpretations," *NeuroImage*, vol. 52, pp. 1059–69, 2010.

[7] T. Schieber, L. Carpi, A. Dìaz-Guilera, P. Pardalos, C. Masoller, and M. Ravetti, "Quantification of network structural dissimilarities," *Nature Communications*, vol. 8, p. 13928, 2017.
